# Electronic Structure and Chemical Bonding of the First-, Second-, and Third-Row-Transition-Metal Monoborides: The Formation of Quadruple Bonds in RhB, RuB, and TcB

**DOI:** 10.3390/molecules28248016

**Published:** 2023-12-08

**Authors:** Constantinos Demetriou, Christina Eleftheria Tzeliou, Alexandros Androutsopoulos, Demeter Tzeli

**Affiliations:** 1 Laboratory of Physical Chemistry, Department of Chemistry, National and Kapodistrian University of Athens, Panepistimiopolis Zografou, 157 84 Athens, Greece; costasdim@chem.uoa.gr (C.D.); ctzeliou@chem.uoa.gr (C.E.T.); aandrou@chem.uoa.gr (A.A.); 2 Theoretical and Physical Chemistry Institute, National Hellenic Research Foundation, 48 Vassileos Constantinou Ave., 116 35 Athens, Greece

**Keywords:** calculations, DFT, transition metal, borides, ScB, TiB, VB, CrB, MnB, FeB, CoB, NiB, CuB, ZnB, YB, ZrB, NbB, MoB, TcB, RuB, RhB, PdB, AgB, CdB, LaB, HfB, TaB, WB, ReB, OsB, IrB, PtB, AuB, HgB

## Abstract

Boron presents an important role in chemistry, biology, and materials science. Diatomic transition-metal borides (MBs) are the building blocks of many complexes and materials, and they present unique electronic structures with interesting and peculiar properties and a variety of bonding schemes which are analyzed here. In the first part of this paper, we present a review on the available experimental and theoretical studies on the first-row-transition-metal borides, i.e., ScB, TiB, VB, CrB, MnB, FeB, CoB, NiB, CuB, and ZnB; the second-row-transition-metal borides, i.e., YB, ZrB, NbB, MoB, TcB, RuB, RhB, PdB, AgB, and CdB; and the third-row-transition-metal borides, i.e., LaB, HfB, TaB, WB, ReB, OsB, IrB, PtB, AuB, and HgB. Consequently, in the second part, the second- and third-row MBs are studied via DFT calculations using the B3LYP, TPSSh, and MN15 functionals and, in some cases, via multi-reference methods, MRCISD+Q, in conjunction with the aug-cc-pVQZ-PP_M_/aug-cc-pVQZ_B_ basis sets. Specifically, bond distances, dissociation energies, frequencies, dipole moments, and natural NPA charges are reported. Comparisons between MB molecules along the three rows are presented, and their differences and similarities are analyzed. The bonding of the diatomic borides is also described; it is found that, apart from RhB(*X*^1^Σ^+^), which was just recently found to form quadruple bonds, RuB(*X*^2^Δ) and TcB(*X*^3^Σ^−^) also form quadruple *σ*^2^*σ*^2^*π*^2^*π*^2^ bonds in their X states. Moreover, to fill the gap existing in the current literature, here, we calculate the TcB molecule.

## 1. Introduction

Boron has an important role in chemistry, biology, and materials science [1]. It is well known that it forms single, double, and triple bonds, but it was only recently found that it can form quadruple bonds in specific diatomic molecules [2,3,4,5]. Additionally, its chemistry is quite interesting to preparative chemists, theoreticians, industrial chemists, and technologists. It is noteworthy that it is the only non-metal in group 13 of the periodic table, and it presents many similarities to its neighbor, carbon, and its diagonal relative, silicon. Hence, like C and Si, it showcases a marked propensity to form covalent, molecular compounds, but it differs greatly from them in having one less valence electron, a situation sometimes referred to as “electron deficiency”. This deficiency plays a key role in its chemistry [1].

Transition-metal borides have received considerable attention since they present common catalytic properties for the hydrogenation of alkenes and alkynes, the reduction of nitrogenous functional groups, and deoxygenation reactions [6]. They are important building blocks in many complexes and materials. Moreover, they possess remarkable physical properties, such as very high conductivity (TiB_2_) [7]—even superconductivity (MgB_2_) [8]—as well as super hardness (ReB_2_) [9]. In solid state, many computational and experimental studies have been carried out; see, for instance, [10,11,12,13,14,15,16,17,18]. Computationally, the DFT methodology is applied to determine the bond lengths, frequencies, and vibrational properties of solids [10,13]; density of state; bond population; charge density maps [14]; relative stability; mechanical, electronic, and magnetic properties [15]; elastic behavior; and elastic anisotropy [16]. Furthermore, ab initio molecular dynamic (AIMD) simulations at finite temperature have also been employed in order to investigate the structural stability of materials, for instance, those of Ʋ_2_B (Ʋ = Ti, Cr, Nb, Mo, Ta, and W) [18].

It has been reported that the electronic structure and the chemical bonding of diatomic and triatomic molecules are strongly related to their structure, the variety of their morphologies, and the properties of their 2D materials and solid state [19,20]. Therefore, an investigation of the electronic structure and the bonding of the diatomic transition-metal borides, which constitute the simplest building blocks of the compounds or materials in question, would lay the foundation for understanding the very complex solid-metal borides and even their bulk properties. Finally, it should be noted that the diatomic transition-metal borides showcase unique electronic structures presenting interesting and peculiar properties and a variety of bonding schemes.

The present work has two aims. In the first part, we present a review of the experimental and theoretical studies on the first-row-transition-metal borides, i.e., ScB, TiB, VB, CrB, MnB, FeB, CoB, NiB, CuB, and ZnB; the second-row-transition-metal borides, i.e., YB, ZrB, NbB, MoB, TcB, RuB, RhB, PdB, AgB, and CdB; and the third-row-transition-metal borides, i.e., LaB, HfB, TaB, WB, ReB, OsB, IrB, PtB, AuB, and HgB. In the second part, the second- and third-row-transition-metal borides, MBs, are studied via DFT calculations using the B3LYP, TPSSh, and MN15 functionals in conjunction with the aug-cc-pVQZ-PP_M_/aug-cc-pVQZ_B_ basis sets. Additionally, MRCISD(+Q) calculations were carried out to clarify the ground states of the MB molecules when their identity was not known. Bond distances, dissociation energies, frequencies, dipole moments, and Mulliken and natural NPA charges are presented. Comparisons between the MB molecules of all three rows are presented, and their differences and similarities are analyzed. Finally, transition-metal borides forming quadruple bonds are described and analyzed here, and for the first time, we report on the RuB and TcB molecules.

## 2. Previous Studies on Transition-Metal Monoborides, MBs

### 2.1. First-Row-Transition-Metal MBs

All previous theoretical and experimental data on the ground states of the first-row-transition-metal borides are summarized in Table 1 [21,22,23,24,25,26,27,28,29,30]. The first study of each first-row-transition-metal boride (MB) was carried out by Wu in 2005, who studied the MB molecules via DFT methodology, i.e., B3LYP/6-311++G(3df) [22]. In 2008, Tzeli and Mavridis, using multireference methods (MRCI), systematically studied the electronic structure and bonding of the ground and some low-lying states, up to twenty-four excited states, of all first-row-transition-metal borides (MBs). They used multireference methods employing correlation-consistent basis sets of quintuple cardinality (cc-pV5Z) [21]. Full potential-energy curves were constructed at the MRCI/cc-pV5Z level for the lowest up to five states, while about twenty states for every MB species were examined at the MRCI/cc-pVQZ_B_ANO-4Z_M_ level of theory. At the MRCI/cc-pV5Z level, total energies, dissociation energies, dipole moments, and common spectroscopic parameters of the nine diatomic borides, MBs, M = Sc–Cu, were reported. Ground states of the MBs along with “recommended” bond distances, dissociation energies, and dipole moments were calculated, and boron atoms’ exceptional ability to participate in a variety of bonding schemes was stressed; the bonding in these MB series varied from three half bonds to full triple bonds. Furthermore, the core correlation using the cc-pwCV5Z basis set was calculated for specific molecules as well as the Scalar relativistic effects through the second-order Douglas–Kroll–Hess approximation, i.e., at C-MRCI+DKH2/cc-pwCV5Z-DK. This study [21], using very accurate methodology, calculated bond distances, dissociation energies, dipole moments, and spectroscopic parameters in excellent agreement with experimental studies which were subsequently conducted the following decade; see Table 1 and discussion below.

**ScB**: In 2008, the electronic structure and bonding of the ground and some low-lying states of ScB were calculated employing MRCI methodology, including the scalar relativistic effects and the correlation of the core electrons [21]. The ground state, *X*^5^Σ^–^, was calculated at the C-MRCISD+DKH+Q/cc-pV5Z level of theory, and a value of 2.094 Å was found for the bond length, 3.309 eV was found for the dissociation energy with respect to the adiabatic atomic products, and 603 cm^−1^ was found for the vibrational frequency. In 2021, Merriles et al. performed resonant two-photon ionization spectroscopy (R2PI) experiments to measure the predissociation threshold in ScB and found a value of *D*_0_ (ScB) = 1.72(6) eV with respect to the ground state products [25], while the theoretical dissociation energy with respect to the atomic ground state was 1.74 eV [21], in excellent agreement with the experimental value of 1.72(6) eV [25]. The bonding in the ground state consists of three half bonds [21].

**TiB**: The ground state of TiB is the *X*^6^Δ state. Its bond distance was calculated to be 2.080 Å with a dissociation energy of 2.797 eV and a vibrational frequency of 583 cm^−1^ using icMRCISD/cc-pV5Z [21]. Recently, Merriles et al. measured the predissociation threshold in TiB to be *D*_0_ (TiB) = 1.956(16) eV via R2PI spectroscopy [25]. Finally, in the ground state, three half bonds are formed.

**VB**: The its ground state of VB, *X*^7^Σ^+^, presents similar bonding behavior to the ground state of the ScB and TiB molecules, i.e., three half bonds are formed [21]. Its bond distance is 2.043 Å, with a dissociation energy, *D*_e_, of 2.381 eV with respect to the adiabatic products, and 2.143 eV with respect to the atomic ground state products, while the vibrational frequency, *ω*_e_, is 589.9 cm^−1^ [21]. The R2PI predissociation threshold in VB, *D_0_*, was measured to be 2.150(16) eV [25], in excellent agreement with the theoretical value.

**CrB**: The bonding of the CrB ground state, *X*^6^Σ^+^, is a *σ*^2^ bond and two half *π* bonds, i.e., *σ*^2^*π*^1^*π*^1^ [21]. Using the icMRCISD+Q/cc-pV5Z methodology, the Cr-B distance was calculated to be 2.183 Å with a binding energy of 1.353 eV, a rather small dissociation energy, while the *ω*_e_ value was 405 cm^−1^. The B3LYP bond distance, 2.187 Å [22], is in very good agreement with the corresponding value obtained using the icMRCISD+Q/cc-pV5Z methodology [21].

**MnB**: The relative ordering of twenty-six states of MnB has been calculated [21]. The ground state is the *X*^5^Π state, with a value of 2.183 Å for the bond length, 0.854 eV for the dissociation energy, and 391.9 cm^−1^ for the vibrational frequency. The bonding is *σ*^2^*π*^1^*π*^1^; however, the dissociation energy is very small. On the contrary, the first excited state, *A*^5^Σ^−^, which lies 0.09 eV above the *X* state, has three bonds, *σ*^2^*π*^2^*π*^2^, a short bond length of 1.832 Å, and a significant larger dissociation energy of 3.131 eV than the *X* state obtained using the icMRCISD+Q/cc-pV5Z methodology [21]. Note that in the *A*^5^Σ^−^ state, Mn is excited at its ^5^D atomic state.

**FeB**: In 2005, the ground state, ^4^Σ^−^, of the FeB molecule was calculated at the DFT(B3LYP)/6-311++G(3df) level [22]. Then, in 2008, nineteen electronic states were calculated using MRCI, while the electronic structure and chemical bonding of the ground and the first excited states were examined [21]. At the icMRCISD+Q/cc-pV5Z level of theory, the *X*^4^Σ^−^ ground state values are *r*_e_ = 1.743 Å and *D*_e_ = 2.346 eV. In 2019, Merriles et al. measured the predissociation threshold in FeB via R2PI spectroscopy and found a value of *D_0_* = 2.43(2) eV [26]. This work included the first experimental measurement of the BDE of FeB. 

**CoB**: The ground state of CoB, *X*^3^Δ, was calculated in 2005 via the DFT methodology [26], and in 2008, it was calculated using icMRCISD+Q/cc-pV5Z [21]. Eighteen states were calculated. In the ground state, a triple bond is formed with a bond distance of 1.700 Å and a binding energy of 2.849 eV. In 2011, Ng et al. observed and analyzed the electronic transition spectrum of CoB in the visible region between 495 and 560 nm using laser-induced fluorescence spectroscopy [27]. The ground state of CoB was identified to be *X*^3^Δ_3_ with *r*_e_ = 1.705 Å. The ground state’s identity was reconfirmed to be *X*^3^Δ_3_ by Dore et al. [31]. In 2019, Merriles et al. performed resonant two-photon ionization spectroscopy experiments to measure the predissociation threshold of CoB, obtaining a value of *D*_0_ = 2.954(3) eV [26].

**NiB**: The *X*^2^Σ^+^ state of NiB is the ground state, presenting a triple bond. It was calculated via DFT methodology in 2005 [22] and the MRCI method in 2008 [21]. Via MRCI, twenty states were studied. At the icMRCISD+Q/cc-pV5Z level, its bond length was calculated to be 1.681 Å with a binding energy of 3.434 eV and *ω*_e_ = 803 cm^−1^. Also in 2008, Balfour et al. characterized NiB spectroscopically using laser-induced fluorescence spectroscopy [28]. The ground state of NiB was identified to be *X*^2^Σ^+^ with an electronic configuration of 1*σ*^2^2*σ*^2^1*π*^4^1*δ*^4^3*σ*^1^, *r*_e_ = 1.698 Å, *ω*_e_ = 778.0 cm^−1^, and *ω*_e_*x*_e_ = 4.90 cm^−1^ [28]. In 2010, Zhen et al. investigated NiB using LIF spectroscopy to resolve the rotational structure of a band belonging to a newly discovered band system with a ^2^Π_3/2_ upper state [32]. In 2015, Goudreau et al. [33] investigated the 0-0, 2-0, and 3-0 bands of NiB belonging to the ^2^Π^3/2^ *← X*^2^Σ^+^ band system assigned by Zhen et al. [32] at high resolution. The fine structure splitting in each state was determined for the first time, confirming the assignment of the ground state as ^2^Σ^+^ with an electronic configuration of 1*σ*^2^2*σ*^2^1*π*^4^1*δ*^4^3*σ*^1^. Finally, in 2019, Merriles et al., via R2PI spectroscopy, measured the predissociation threshold in NiB to be *D*_0 =_ 3.431(4) eV [26].

**CuB**: In 1997, Barysz and Urban investigated the spectroscopic constants and dipole moment curves of the ground states, *Χ*^1^Σ^+^, of the coinage metal diatomic molecules with boron, i.e., BCu, using high-level-correlated methods combined with quasi-relativistic Douglas–Kroll (no-pair) spin-averaged approximation [29]. At the CCSD(T)/[9s7p3d2f/_Cu_5s3p2d/_B_] computational level, the values *r*_e_ = 1.909 Å, *D*_e_ = 1.522 eV, and *ω*_e_ = 555.0 cm^−1^ were found. In 2005, Wu also studied the *X* state via DFT [22], while in 2008, Tzeli and Mavridis investigated nineteen states using the MRCI+Q methodology [21]. At the icMRCISD+Q/cc-pV5Z level of theory, they found values of *r*_e_ = 1.922 Å, *D*_e_ = 2.129 eV, and *ω*_e_ = 553 cm^−1^. The *D*_e_ value at the icMRCISD/cc-pV5Z level was significantly larger than the corresponding values at the CCSD(T)/[9s7p3d2f/_Cu_5s3p2d/_B_] level due to its significantly larger basis set. In 2023, Merriles and Morse studied CuB experimentally for the first time via R2PI spectroscopy and obtained the first BDE measurement for this molecule. They found that CuB remains bound at energies that far surpass its bond dissociation energy (BDE), and bonds break only when excited at or above an excited sharp predissociation threshold (SAL). Nevertheless, a BDE value of *D*_0_ = 2.26(15) eV was derived [30], which was in very good agreement with the calculated value of 2.129 eV using icMRCISD+Q/cc-pV5Z [21].

**ZnB**: Only one theoretical study was found for ZnB. Its ground state, *Χ*^2^Π, was calculated via the DFT methodology, B3LYP/6-311++G(3df) [22], obtaining a value of *r*_e_ = 2.274 Å with a very small binding energy of 0.370 eV. Here, we calculated the X state at the B3LYP, TPSSh, and MN15/aug-cc-pVQZ(-PP) levels of theory. Both B3LYP and TPSSh provided the same D_e_ values, i.e., 0.373 eV, while TPSSh overestimated it. Here, we found that the B3LYP/aug-cc-pVQZ(-PP) methodology is in very good agreement with the available experimental data on MBs compared with the other functionals. Thus, we consider it to be our best DFT methodology. The bond distance was found to be 2.258 Å and the dipole moment was found at 1.65 D.

### 2.2. Second-Row-Transition-Metal MBs

All previous theoretical and experimental data on the ground states of the diatomic metal borides including the second-row transition metals are summarized in Table 2. Below, they are discussed in detail. There is no experimental or theoretical study on TcB except a D_0_ value for the ^5^Σ^−^, obtained via DFT(B97-1). Note that Tc is a synthetic element, and all its isotopes are radioactive. In this paper, we fill this gap by studying the TcB molecule.

**YB**: The first report on YB was in 1969 by K. A. Gingerich [24], who estimated the dissociation energy of the molecule to be 2.99 eV using the Pauling method of electronegativities [24,25]. In 2009, Kharat et al. calculated the ground spin states of the second-row (4d) transition metals (except for Tc) and their cationic and anionic counterparts at the DFT(B3LYP)/LANL2DZ level. They calculated the bond distances, *r_e_*; binding energies, *D_e_*; electron affinities (EA); ionization potentials (IP); vibrational frequencies, *ω_e_*; and dipole moments, *μ* [34]. For the diatomic YB, a quintet (*S* = 2) ground spin state was established. The report lacks details about its electronic configuration, and as such, its ground spin state electronic symmetry is not included. The associated bond distance was found to be 2.254 Å, the binding energy was 2.17 eV, the EA was 0.69 eV, the IP was 6.16 eV, *ω_e_* = 582.4 cm^−1^, and *μ* = 4.65 D. The most recent investigation into the electronic structure of the YB dimer was made in 2021 by Merriles et al. [25]. They measured the predissociation thresholds of several early-transition-metal boride diatomics using resonant two-photon ionization (R2PI) spectroscopy. For the YB molecule, a *D*_0_ value of 2.057(3) eV was obtained. Additionally, they provided an insight into the chemical bonding and electronic structures of those same species by performing quantum chemical calculations using the DFT (B97-1) methodology. Computational results showed excellent agreement with the measurements for YB, and its ground state was computed to be the wavefunction *X*^5^Σ^−^ (with a dominating 1*σ*^2^2*σ*^1^3*σ*^1^1*π*^2^ configuration determinant), resulting in a bond distance *r*_e_ of 2.306 Å, a dissociation energy *D*_0_ of 1.96 eV, and *ω_e_* = 517 cm^−1^.

**ZrB**: This molecule has only been studied together with other similar species, once in 2009 by Kharat et al. [34] and in 2021 by Merriles et al. [25]. In the first study, a sextet (*S* = 5/2) ground spin state was determined, with r_e_ = 2.189 Å, D_e_ =3.92 eV, EA = 0.48 eV, IP = 7.03 eV, ω_e_ = 582.4 cm^−1^, and μ = 3.48 D. In the latter, the predissociation threshold revealed a *D*_0_ value of 2.573(5) eV, and the ground electronic spin state corresponded to the *X*^6^Δ symmetry wavefunction (with a dominating 1*σ*^2^2*σ*^1^3*σ*^1^1*π*^2^1*δ*^1^ configuration determinant), resulting in a 2.159 Å bond distance, a 2.61 eV dissociation energy, *D*_0_, and an *ω*_e_ value of 610 cm^−1^.

**NbB**: Similarly to the previous molecule, there are two studies on NbB [25,34]. In the first study [34], a triplet (*S* = 1) ground spin state was found, with a bond distance of 1.996 Å, a binding energy of 3.40 eV, EA = 1.05 eV, IP = 7.03 eV, *ω*_e_ = 662.7 cm^−1^, and *μ* = 3.84 D. In the latter [25], the predissociation threshold revealed a *D*_0_ value of 2.989(12) eV, and the computed ground spin state corresponded to the superposition *X*^5^Π/^5^Φ (with a 1*σ*^2^2*σ*^1^3*σ*^1^1*π*^3^1*δ*^1^ configuration determinant), due to the calculations using real forms of the 1*π* and 1*δ* orbitals, providing a bond distance of 1.988 Å, a dissociation energy of 3.07 eV, and a vibrational frequency of 698 cm^−1^. Here, we carried out MRCISD(+Q)/aug-cc-PVQZ(-PP) calculations, and we clarified that the X state is the *X*^5^Π state, while the *A*^5^Φ is located 0.084(0.093) eV above the X state; see discussion below. 

**MoB**: In the first report of MoB [34], it was claimed that the ground state is doublet (*S* = 1/2), with a 1.817 Å bond distance, a 6.40 eV binding energy, EA = 0.21 eV, IP = 8.68 eV, *ω*_e_ = 826 cm^−1^, and *μ* = 4.05 D. In 2011, Borin and Gobbo [35] investigated the electronic structure of the Χ state and the low-lying electronic states of MoB and its cationic MoB^+^ counterpart by employing quantum computational CASSCF protocols on the CASPT2+DKH/4*ζ*-8*s*7*p*5*d*3*f*2*g*-ANO-RCC_Mo_/4*ζ*-5*s*4*p*3*d*2*f*_B_ level. MoB’s ground spin state was computed to be the wavefunction X^6^Π (with a dominant 1*σ*^2^2*σ*^1^3*σ*^1^1*δ*^2^1*π*^3^ configuration determinant) with a bond distance, *r*_e_, of 1.968 Å, a vibrational frequency, *ω*_e_, of 664 cm^−1^, and a dipole moment, *μ*, of 2.7 D. The binding energy was also determined to be 2.18 eV.

**TcB**: TcB is the least studied molecule. Its only study resulted in a *D*_0_ value calculated to be 3.31 eV [25] via the B97-1/(aug)-cc-pVTZ-(PP) methodology for the *X*^5^Σ^−^ state. Tc is the lightest element, and all its isotopes are radioactive. In this paper, we fill this research gap, and we study three states of the TcB molecule. The main spectroscopic data are provided; see discussion below and tables of Section 3 below. Here, we found that the ground state is an *X*^3^Σ^−^ state which presents a quadruple bond; see discussion below.

**RuB**: This molecule was studied experimentally via mass spectrometry for the first time by Auwera-Mahieu et al. in 1970 [36]. Via the Knudsen effusion method, they determined its dissociation energy, *D*_0_, to be 4.59(22) eV. The *ω_e_* value of 915 cm^−1^ was estimated from the values of the corresponding carbides using the *D*_0_(A)/*D*_0_(B) = *μ*_A_*ω*_A_^2^/*μ*_B_*ω*_B_^2^ equation, where *μ* is the reduced mass. The internuclear distance was estimated from the values of the carbides using the formula *r*_MB_ = *r*_MC_ + ½ (*r*_B₂_ − *r*_C₂_), resulting in a value of 1.75 Å. Finally, they proposed that the X state is a ^2^Σ state. It took nearly four decades for this molecule to be inspected again, and in the 2009 work of Kharat et al. [34], a doublet (*S* = ½) ground spin state was reported for the diatomic RuB, with a bond distance of 1.761 Å, a binding energy of 6.48 eV, an *EA* of 0.35 eV, an *IP* of 9.06 eV, an *ω*_e_ of 910.8 cm^−1^, and a dipole moment of 3.49 D. In 2012, Wang et al. [37] studied the laser-induced fluorescence spectrum of RuB in the visible region between 500 nm and 575 nm. They determined that the ground state symmetry is *X*^2^Δ, consistent with an electronic configuration obtained using molecular orbital energy level diagrams, while the bond length, *r*_0_, is 1.7099 Å. In 2019, Merriles et al. [26] used R2PI spectroscopy and accurately assigned the bond dissociation energies of the diatomic late-transition-metal monoborides from the measurement of a predissociation threshold. The measured predissociation threshold resulted in *D*_0_ = 4.815(3) eV. Continuing their work, in 2022, Merriles et al. [45] investigated the ionization energies and the cationic dissociation energies of the diatomic second- and third-row-late-transition-metal borides they had previously examined. Resonant two-photon ionization spectroscopy was employed, and the ionization threshold of RuB was measured to be 7.879(9) eV. Regarding the ground state, it was characterized as *X*^2^Σ via Knudsen effusion [36] and ^2^Δ_5/2_ via LIF spectroscopy. Here, we clarify that the *X*^2^Δ state is the ground state, and that it presents a quadruple bonding; see discussion in the next section. 

**RhB**: In 1970, Auwera-Mahieu et al. [36], via the Knusden effusion method, yielded a value of 4.89(22) eV for the *D*_0_, dissociation energy, of RhB. The vibrational frequency, the bond distance, and the ground state were proposed to be 915 cm^−1^, 1.75 Å, and ^1^Σ, respectively. In 2006, Chowdhury and Balfour [38] measured the gas phase electronic spectrum of RhB in the visible region; it was elucidated that the ground electronic state is of *X*^1^Σ^+^ symmetry, with an internuclear distance of 1.691(2) Å. The following year, in 2007, Gobbo and Borin [39] studied the low-lying ^1^Σ^+^ states of RhB at the CASSCF/MS-CASPT2/4*ζ*-ANO-RCC_Rh_/14*s*9*p*4*d*3*f*_B_ level to answer some questions raised by the previous experiments. In agreement with the experiment, their results indicated that the ground electronic state corresponded to the X^1^Σ^+^ wavefunction (with a dominant 1*σ*^2^2*σ*^2^1*π*^4^1*δ*^4^ configuration determinant) with an internuclear distance, *r*_0_, of 1.698 Å. In the same year, another study by Chowdhury and Balfour [40] resumed their previous spectroscopic studies, with a clear focus on the intrinsic details of the emerging bands. In 2008, A.C. Borin and J.P. Gobbo [41], in order to gain further insight into the structural and spectroscopic properties of RhB, investigated its first two atomic dissociation channels. The first regarded the adiabatic coupling of the two atoms in their ground atomic states, B(2*s*^2^2*p*;^2^P) and Rh(4*d*^8^(^3^F)5*s*;^4^F), while in the second, the rhodium atom participated in its first excited atomic electronic state, Rh(4*d*^9^;^2^D), at the CASSCF/MS-CASPT2/4*ζ*-ANO-RCC_Rh_/14*s*9*p*4*d*3*f*_B_ level of theory. Results showcased that *X*^1^Σ^+^ correlates with the second atomic dissociation limit. The researchers predicted a 5.6 eV dissociation energy, *D*_0_; a 924 cm^−1^ vibrational frequency, *ω*_e_; and a dipole moment of 4.54 D. The Mulliken population analysis yielded a charge of +0.35*e* on Rh. In the 2009 work of Kharat et al. [34], a singlet (*S* = 0) ground state was reported for RhB, with a 1.745 Å bond distance, a 6.48 eV binding energy, EA = 0.85 eV, IP = 8.19 eV, and *μ* = 2.84 D. Later, in the 2019 work by Merriles et al. [26], a predissociation threshold of *D*_0_ = 5.252(3) eV was measured with the use of R2PI spectroscopy. In 2020, two works concerning the bonding structure of RhB were published, where the formation of a quadruple bond was reported. The first study conducted by Cheung et al. [2] explored the bonding nature of RhB(BO)^−^ and RhB species. With the use of PES, an electronic fingerprint was obtained, and the electron affinity of the dimer was measured experimentally to be 0.961 eV. In all computational levels, ADF, DFT, CCSD(T), it was found that the electronic ground state corresponds to an *X*^1^Σ^+^ wavefunction (with a dominant 1*σ*^2^1*π*^4^2*σ*^2^1*δ*^4^ configuration determinant), resulting in a quadruple bond consisting of two *π* bonds formed between the Rh 4*d_xz_*/4*d_yz_* and B 2*p_x_*/2*p_y_* orbitals and two *σ* bonds between the Rh 4*d_z_*_²_ and B 2*s*/2*p_z_* orbitals, followed by internuclear distances ranging from 1.685 Å to 1.689 Å. At the ADF/PBE/TZ2P level, it was also possible to obtain a 5.27 eV value for the dissociation energy. The second study, carried out by Tzeli and Karapetsas [4], investigated the bond occurring inside isoelectronic species between transition metals and the main group elements TcN, RuC, RhB and PdBe. For the RhB molecule, at various levels of theory, i.e., MRCISD, MRCISD+Q, and RCCSD(T)/aug-cc-pV5Z-PP_Rh_ aug-cc-pV5Z_B_, the common spectroscopic constants were computed, presenting great agreement among themselves, as well as with the experimental values. Specifically, values of *r*_e_ =1.6872 Å, *D*_e_ = 5.490 eV, and *ω*_e_ = 942.1 cm^−1^ were found, along with anharmonic corrections of *ω*_e_*x*_e_ =3.78 cm^−1^ and *μ* = 2.965 D [4]. It was deduced that the ground electronic state (*X*^1^Σ^+^) has a dominant 1*σ*^2^2*σ*^2^1*π*^4^1*δ*^4^ configuration determinant and correlates adiabatically to the atomic electronic spin states B(*X*^2^P;2*s*^2^2*p*) + Rh(*a*^2^D;4*d*^9^), resulting in a four-fold quadruple bond. Tzeli and Karapetsas [4] found that, except for the ground state of RhB, its two lowest excited states, i.e., *a*^3^Δ and A^1^Δ, also present quadruple bonds [4]. Additionally, in the ground and the first excited states of the RhB^−^ anion, *Χ*^2^Σ^+^ and A^2^Δ quadruple bonds are also formed [5]. In 2021, Schoendorff et al. [42] also studied the bonding in RhB both qualitatively and quantitively at a MCSCF-IOTC/DKH-TZP-2012_Rh_/Sapporo-TZP-2012_B_ level of theory and reached the same results as those in previous works. They concluded that the ground electronic spin state corresponded to the *X*^1^Σ^+^ symmetry wavefunction (with a dominant 1*σ*^2^2*σ*^2^1*π*^4^1*δ*^4^ configuration determinant), with an equilibrium bond length of 1.701 Å and a binding energy of 5.165 eV. Finally, in 2022, Merriles and Morse [45] measured the ionization potential and obtained a value of 8.234(10) eV.

**PdB**: Via the Knusden effusion method, the dissociation energy, *D*_0_, of PdB was measured to be 4.89(22) eV, with *ω* = 650 cm^−1^ and a bond distance of 2.00 Å, while the proposed ground state was ^2^Σ [36]. In 1992, Knight Jr. et al. [43], via electron spin resonance (ESR) spectroscopy, revealed that the ground electronic state of the dimer is *X*^2^Σ. Unrestricted Hartree–Fock (UHF) calculations were also carried out and a 1.608 Å internuclear distance was deduced. In 2009, a DFT study [34] predicted a doublet (*S* = ½) ground state with a bond distance of 1.856 Å and a binding energy of 3.33 eV. The most recent study on PdB was published in 2012 by Ng et al. [44], marking its first electronic spectroscopic investigation using laser-induced fluorescence spectroscopy in the visible region between 465 and 520 nm. An *X*^2^Σ^+^ ground state was revealed, with *r*_0_ = 1.7278 Å. Moreover, a molecular-orbital-energy-level diagram was designed to understand the observed ground state, and the proposed configuration was determined to be 1*σ*^2^2*σ*^2^1*π*^4^1*δ*^4^3*σ*^1^.

**AgB**: In 1997, Barysz and Urban [29] investigated the spectroscopic constants of AgB at many levels of theory and the plotted dipole moment curves of AgB using high-level-correlated methods combined with quasi-relativistic Douglas–Kroll (no-pair) spin-averaged approximation. The obtained ground state was *X*^1^Σ^+^ in all of them. At the DK-CCSD(T)-20/NpPolMe level of theory, a bond length of 2.115 Å and a dissociation energy of 0.910 eV were obtained, while the corresponding values at the DK-CASPT2/NpPolMe level were 2.098 Å and 1.684 eV. It was highly advised that both relativistic as well as correlation effects be carefully considered to obtain accurate results. Note that due to the relativistic shrinkage of *s* valence shell electrons, stronger bonds are formed which would, otherwise, not be described successfully. Finally, Kharat et al. [34] predicted via DFT a singlet (*S* = 0) ground state with a 2.187 Å bond distance and a 1.60 eV binding energy.

**CdB**: There is only one DFT study in the literature. Kharat et al. [34] reported a doublet (*S* = ½) ground state with a 2.668 Å bond distance, a 0.22 eV binding energy, an EA of 0.09 eV, an *IP* of 7.05 eV, *ω*_e_ = 198.3 cm^−1^, and *μ* = 1.67 D.

### 2.3. Third-Row-Transition-Metal MBs

Table 3 summarizes the previous experimental and theoretical data obtained for LaB, HfB, TaB, WB, ReB, OsB, IrB, PtB, AuB, and HgB. The calculations are mainly DFT apart from those of HfB [46], PtB [47], and AuB [29,47], for which CCSD(T), CASPT2, and MRCI calculations were carried out.

**LaB**: In 1969, K. A. Gingerich [24] estimated the binding energy at 3.51 eV. In 2010, Kalamse et al. [48] studied the 5*d*-metal mononitrides and monoborides using DFT methodology with the B3LYP functional set and both LANL2DZ and SDD basis sets. The lowest electronic spin states at these two DFT levels of theory, as well as *EA*, *IP*, binding energies, and electronic configurations of the MBs, were discussed, while the orbitals involved in bond formation were identified. At the B3LYP/LANL2DZ level, it was deduced that the most stable state for LaB is *X*^5^Σ*^–^*, with a bond length of 2.435 Å, a binding energy of 2.49 eV, *μ* = 4.29 D, EA = 1.01 eV, and IP = 5.61 eV. On the other hand, for the B3LYP/SDD level, the most stable state was found to be *X*^3^Π, with *r*_e_ = 2.336 Å and *D*_e_ = 2.49 eV. In 2018, Elkahwagy et al. [49] studied LaB and its anionic LaB^−^ counterpart with the diffusion Monte Carlo method in combination with three different trial functions to calculate the potential energy curves for the lowest electronic states of those species, along with some spectroscopic constants of neutral LaB. Irrespectively of the functional used, it was found that the quintet state of LaB is the ground state, while the triplet state is higher in energy, elucidating the mystery that the previous work arose. The 2021 study by Merriles et al. [25] also suggested that the ground spin state corresponded to an *X*^5^Σ^−^ symmetry wavefunction (with a dominant 2*σ*^1^3*σ*^1^1*π*^2^ configuration determinant) with a dissociation energy, *D*_0_, of 2.54 eV; a bond length, *r*_e_, of 2.372 Å; and a vibrational frequency, *ω*_e_, of 521 cm^−1^. The experimental part of their study yielded a 2.086(18) eV value for the dissociation energy from the predissociation threshold. Here, both the lowest triplet and quintet states were calculated, and we found that the ground one is the *X*^5^Σ^−^ state.

**Table 3:** Previous theoretical and experimental data on the ground states of the 3rd-row-transition-metal boride molecules, MBs (M = La, Hf, Ta, W, Re, Os, Ir, Pt, Au, and Hg): bond lengths *r*_e_ (Å), dissociation energies *D*_e_ (eV) and/or *D*_0_ (eV), electron affinities *EA* (eV), ionization potentials *IP* (eV), vibrational frequencies *ω*_e_ (cm^−1^), anharmonic corrections *ω*_e_*x*_e_ (cm^−1^), and dipole moments *μ* (*μ*_FF_ = *δE*/*δε*) (Debye).

MB	Methodology	Ref.	State	*r*_e_	*D*_e_ ^a^	*D*_0_	*ω*_e_	*ω*_e_ *x*_e_	*μ* (*μ*_FF_)
**LaB**	DFT: B3LYP/LANL2DZ	[48]	*X*^5^Σ^−^	2.435	2.49		511		4.29
	DFT/B3LYP/SDD	[48]	*X*^3^Π	2.336	2.10		496		5.02
	DMC(B3LYP)/CRENBS-ECP_La_Burkatzki-PP_B_	[49]	*S* = 2	2.150	3.37				4.22
	DMC(B3PW91)/CRENBS-ECP_La_ Burk.-PP_B_	[49]	*S* = 2	2.145	3.81				4.19
	R2PI spectroscopy	[25]				2.086(18)			
	DFT: UB97-1/AVTZ-PP_La_ VTZ_B_	[25]	*X*^5^Σ^−^	2.372	2.57	2.54	521		
	Pauling method	[24]				3.51			
**HfB**	DFT: B3LYP/LANL2DZ	[48]	*X*^4^Σ*^–^*	2.157	2.70		613		2.44
	DFT/B3LYP/SDD	[48]	*X*^4^Σ*^–^*	2.195	2.64		580		2.68
	R2PI spectroscopy	[25]				2.593(3)			
	DFT: UB97-1/AVTZ-PP_La_ VTZ_B_	[25]	*X*^4^Σ*^–^*	2.128	2.64	2.60	584		
	c-CCSD(*T*)/wCV5Z-PP_Hf_ AV5Z_B_	[46]	*X*^4^Σ^−^	2.144	2.841	2.840	607	2.8	
	MRCI/c-CCSD(*T*)/wCVQZ-PP_Hf_ AVQZ_B_	[46]	*X*^4^Σ^−^	2.174			610	3.0	
	BP86/def2-QZVP	[46]			3.182				
	BLYP/def2-QZVP	[46]			2.803				
	BPE/def2-QZVP	[46]			3.311				
	MN15-L/def2-QZVP	[46]			3.071				
	LRC-ωPBEh/def2-QZVP	[46]			2.174				
	DSD-PBEB95-D3BJ/def2-QZVP	[46]			1.923				
**TaB**	DFT: B3LYP/LANL2DZ	[48]	*X*^3^Σ^+^	2.001	2.49		721		2.68
	DFT/B3LYP/SDD	[48]	*X*^5^Δ	2.184	2.48		555		1.44
	R2PI spectroscopy	[25]				2.700(3)			
	DFT: UB97-1/AVTZ-PP_Ta_ VTZ_B_	[25]	*X*^5^Δ	2.085	3.00	2.95	754		
**WB**	DFT: B3LYP/LANL2DZ	[48]	*X*^6^Σ^−^	2.161	2.74		526		2.61
	DFT/B3LYP/SDD	[48]	*X*^6^Σ^−^	1.990	2.88		725		2.67
	R2PI spectroscopy	[25]				2.730(4)			
	DFT: UB97-1/AVTZ-PP_W_ VTZ_B_	[25]	*X*^6^Σ^+^	1.891	2.94	2.89	730		
**ReB**	DFT: B3LYP/LANL2DZ	[48]	*X*^3^Σ^−^	1.842	2.77		867		2.99
	DFT: B3LYP/SDD	[48]	*X*^5^Σ^−^	1.875	4.18		853		2.29
**OsB**	R2PI spectroscopy	[26]	GS			4.378(3)			
	B3LYP/aug-cc-pVQZ-PP	[45]	*X*^4^Σ^−^	1.770			938		2.24
	B3LYP/LANL2DZ	[48]	*Χ*^4^Δ	1.813	3.99		955		2.17
**IrB**	LIF spectroscopy	[50]	*Χ*^3^Δ_3_	1.7675					
	B2PLYP/AVQZ(-PP)_M_	[47]	*Χ*^3^Δ	1.763					
	CCSD(T)/AVQZ(-PP)	[47]	*Χ*^3^Δ			5.085			
	R2PI spectroscopy	[26]	GS			4.928(10)			
	Mass spectrometry	[36]	GS			5.27(18)			
	B3LYP/LANL2DZ	[48]	*Χ*^3^Δ	1.806	4.86		936		1.72
**PtB**	R2PI spectroscopy	[26]	GS			5.235(3)			
	Mass spectrometry	[51]	GS			4.91(17)			
	LIF spectroscopy	[52]	*Χ*^2^Σ^+^	1.741			903.6		
	B3LYP/LANL2DZ	[48]	*Χ*^2^Σ^+^	1.809	5.43		906		1.18
	B2PLYP/AVQZ(-PP)	[47]	*Χ*^2^Σ^+^	1.755					
	CCSD(T)/AVQZ(-PP)	[47]	*Χ*^2^Σ^+^			5.668			
**AuB**	Mass spectrometry	[36]	GS			3.50(16)			
	Knudsen cell experiment	[53]	GS			3.773			
	B3LYP/LANL2DZ	[48]	*Χ*^1^Σ^+^	1.997	2.96		559		0.68
	B2PLYP/AVQZ(-PP)	[47]	*Χ*^1^Σ^+^	1.906			710		
	CCSD(T)/AVQZ(-PP)	[47]	*Χ*^1^Σ^+^			3.734			
	Nonrelativistic CASPT2/PolMe	[29]	*Χ*^1^Σ^+^	2.256	1.261		362	3.06	
	No-pair DK CASPT2/NpPolMe	[29]	*Χ*^1^Σ^+^	1.931	3.519		676	4.76	
	No-pair DK CCSD(*T*)-20/NpPolMe	[29]	*Χ*^1^Σ^+^	1.960	2.709		663	4.01	
	R2PI spectroscopy	[30]	*Χ*^1^Σ^+^			3.724(3)			
**HgB**	B3LYP/LANL2DZ	[48]	*Χ*^2^Σ^+^	4.381	0.002		19		0.27

^a^ Dissociation energy with respect to the atomic ground state products.

**HfB**: The B3LYP/SDD methodology [48] was used to predict *X*^4^Σ*^–^* as the ground state of HfB with a bond length of 2.195 Å, *μ* = 2.60 D, EA = 1.05 eV, IP = 5.01 eV, and a binding energy of 2.64 eV. The researchers found that, in contrast to the rest of the 5*d*-metal monoborides, it is the 5*d* orbital of the metal that loses an electron in the case of HfB. In the 2021 study by Merriles et al. [25], the authors measured the predissociation threshold, i.e., *D*_0_(HfB) = 2.593(3) eV, and the calculated ground spin state corresponded to the *X*^4^Σ^−^ symmetry wavefunction (with a dominant 2*σ*^1^3*σ*^2^1*π*^2^ configuration determinant) with a bond distance of 2.128 Å, a dissociation energy of 2.60 eV, and a vibrational frequency of 584 cm^−1^ at UB97-1/AVTZ-PP_La_VTZ_B_. In 2022, Ariyarathna et al. [46] conducted a comparative study on the outcomes of several computational methods on the basis of high-level, multi-reference configuration interaction theory and coupled cluster quantum chemical calculations, with large quadruple-*ζ* and quintuple-*ζ* quality-correlation-consistent basis sets, as well as DFT, with numerous exchange-correlation functionals that span multiple rungs of “*Jacob’s ladder*”, through the inspection of HfO and HfB, to investigate their performance on species containing third-row transition metals. Ab initio studies of HfB showed, unanimously, that *X*^4^Σ*^–^* is the ground electronic state, owing to a 1*σ*^2^2*σ*^1^3*σ*^2^1*π*^2^ configuration dominant determinant with a bond length of 2.144 Å, dissociation energy at 2.841 eV, and *ω*_e_ = 607 cm^−1^ at the c-CCSD(T)/wCV5Z-PPHf AV5ZB level. DFT calculations were performed on the closed-shell single-reference ground state to evaluate the errors in the predictions from distinct density functionals. The resulting trends were discussed, plotted, and compared.

**TaB**: DFT calculations predict different states to be the ground state of TaB depending on the methodology. B3LYP/LANL2DZ predicted *X*^3^Σ^+^ to be the ground state, with a 2*π*^4^5*σ*^1^6*σ*^1^ electronic configuration, *r*_e_ = 2.001 Å, and *D*_e_ = 2.49 eV. However, at the B3LYP/SDD level, the calculated ground state was *X*^5^Δ state, with *r*_e_ = 2.184 Å, *μ* = 1.44 D, *ΕA * = 1.43 eV, *ΙP* = 7.35 eV, and *D*_e_ = 2.48 eV [48]. In 2021, Merriles et al. [25] determined the *D*_0_ value to be 2.700(3) eV, and they found that at the UB97-1/AVTZ-PP_Ta_VTZ_B_ level, the ground state corresponded to *X*^5^Δ (with a dominant 2*σ*^1^3*σ*^2^1*π*^2^1*δ*^1^ configuration determinant) with *r*_e_ = 2.085 Å, *D*_e_ = 2.95 eV, and *ω*_e_ = 754 cm^−1^.

**WB**: In WB, as in TaB, DFT calculations predict different states to be the ground state, depending on the methodology. Kalamse et al. [48], at both the B3LYP/LANL2DZ and SDD levels, found that the ground state is *X*^6^Σ^−^, where *r*_e_ = 1.990 Å, *μ* = 2.67 D, and *D*_e_ = 2.88 eV at the B3LYP/SDD level [50]. In 2021, Merriles et al. [25] measured *D*_0_ = 2.730(4) eV and calculated the ground state to be *X*^6^Σ^+^, with a dominant 2*σ*^1^3*σ*^2^1*π*^2^1*δ*^2^ configuration determinant, *r*_e_ = 1.891 Å, *D*_e_ = 2.89 eV, and *ω*_e_ = 730 cm^−1^, using the UB97-1/AVTZ-PP_W_VTZ_B_ methodology [25]. Here, we found that the ground state is the *X*^6^Π state, while the *X*^6^Σ^+^ state lies 0.137 eV above the X state; see tables of the Section 3 and discussion below.

**ReB**: There is only a single theoretical study on the ReB molecule at a DFT level; however, different basis sets predict different states to be the ground state. B3LYP/LANL2DZ predicted the ground state to be *X*^3^Σ^−^, with *r*_e_ = 1.842 Å and *D*_e_ = 2.77 eV, while B3LYP/SDD predicted *X*^5^Σ^−^ to be the ground state, with *r*_e_ = 1.875 Å and *D*_e_ = 4.18 eV [48]. Here, we found that the ground state is *X*^5^Σ^−^, while the *a*^3^Σ^−^ state lies 0.099 eV above the X state; see tables of Section 3 and discussion below.

**OsB:** Kalamse et al., via B3LYP/LANL2DZ and B3LYP/SDD, predicted that the ground state of OsB is the *Χ*^4^Δ state. It corresponds with a *π*^4^*δ*^3^5*σ*^1^6*σ*^1^ electronic configuration, with *r*_e_ = 1.813 Å, *ω*_e_ = 955 cm^−1^, *μ* = 2.17 D, and *D*_e_ = 3.99 eV at the B3LYP/LANL2DZ level [48]. In 2019, Merriles et al. performed R2PI spectroscopy experiments to measure the predissociation threshold, and it was found to be *D*_0_ = 4.378(3) eV [26]. Furthermore, they investigated the electronic ground state at the B3LYP/Def2TZVPP [26] and B3LYP/aug-cc-pVQZ-PP levels [45], and they found that it is a triply bonded 1*σ*^2^2*σ*^2^1*π*^4^1*δ*^2^3*σ*^1^, at the *X*^4^Σ^−^ state, which contradicts with the prediction of Kalamse et al. [48], with a bond distance of 1.770 Å. In 2022, Merriles et al. measured, for the first time, the ionization energy of OsB using resonant two-photon ionization spectroscopy, obtaining a value of *IE* (OsB) = 7.955(9) eV [45].

**IrB:** In 1969, Auwera-Mahieu et al. determined the dissociation energy of IrB to be 5.27(18) eV via a mass spectrometric study at high temperatures [36]. In 2005, Ye et al. investigated IrB using LIF spectroscopy [50]. In this study, the ground state of IrB was found to be *X*^3^Δ_3_, with an electronic configuration of 1*σ*^2^1*π*^4^1*δ*^3^2*σ*^1^ and *r*_e_ = 1.7675 Å. In 2010, using the B3LYP/LANL2DZ methodology, it was also found that the most stable state for IrB is *Χ*^3^Δ raised from a *π*^4^*δ*^3^5*σ*^2^6*σ*^1^ electronic configuration, with *r*_e_ = 1.806 Å, *μ* = 1.72 D, *ΕA* = 5.06 eV, *ΙP* = 4.66 eV, and *D*_e_ = 4.86 eV [48]. In 2011, Pang et al. investigated the electronic transition spectrum of IrB in the spectral region between 400 and 545 nm using LIF spectroscopy [54]. Its ground state was also identified to be *X*^3^Δ_3_ with an electronic configuration of 1*σ*^2^2*σ*^2^*π*^4^*δ*^3^3*σ*^1^. In 2019, Merriles et al., using R2PI spectroscopy, measured the predissociation threshold of IrB, obtaining a value of *D*_0_ = 4.928(10) eV [26]. In 2020, Wang et al. investigated the nature of the chemical bonding in IrB, employing high-resolution photoelectron imaging and theoretical B2PLYP and CCSD(T)/aug-cc-pVQZ(-PP) calculations. They calculated a bond distance of 1.763 Å using B2PLYP and a dissociation energy of 5.085 eV at the CCSD(T) level of theory, in good agreement with the experimental values [47]. In 2022, Merriles and Morse measured, for the first time, the ionization energy of IrB using R2PI spectroscopy, obtaining *IE* = 8.301(15) eV [45].

**PtB:** In 1968, via Knudsen effusion mass spectrometry, McIntyre et al. measured the dissociation energy of gaseous PtB to be 4.91(17) eV [51]. Via the B3LYP/LANL2DZ (B3LYP/SDD) levels of theory, the ground state was assigned to be *Χ*^2^Σ^+^, derived from a *π*^4^5*σ*^2^*δ*^4^6*σ*^1^ electronic configuration, with *r*_e_ = 1.809(1.815) Å and *D*_e_ = 5.43(4.86) eV [48]. In 2012, Ng et al. investigated the optical spectrum of PtB in the visible region between 455 and 520 nm using LIF spectroscopy [52]. The ground state of PtB was identified to be *Χ*^2^Σ^+^, determined using an electronic configuration of 1*σ*^2^2*σ*^2^1*π*^4^1*δ*^4^3*σ*^1^, with *r*_e_ = 1.741 Å and *ω*_e_ = 903.60 cm^−1^. In 2019, Merriles et al. performed R2PI spectroscopy measurements, and they found *D*_0_ = 5.235(3) eV [26]. In 2020, Wang et al. investigated the nature of the chemical bonding in PtB, employing high-resolution photoelectron imaging and theoretical calculations using the B2PLYP and CCSD(T)/aug-cc-pVQZ(-PP) methodologies [47]. They calculated a bond distance of 1.755 Å using B2PLYP and a dissociation energy of 5.668 eV at the CCSD(T) level of theory [47]. In 2022, Merriles and Morse measured, for the first time, the ionization energy of PtB using R2PI spectroscopy, obtaining a value of *IE* = 8.524(10) eV [45].

**AuB**: In 1969, via mass spectrometry at high temperatures, Auwera-Mahieu et al. measured the dissociation energy of AuB to be 3.50(16) eV [36]. In 1971, Gingerich investigated AuB using a combination of Knudsen effusion and mass spectroscopic techniques. The reaction enthalpies determined by the second and third law method yielded *D*_0_ = 3.77 eV [53]. In 1997, Barysz and Urban investigated the spectroscopic constants and dipole moment curves of the AuB ground state, *Χ*^1^Σ^+^, using high-level-correlated methods combined with quasi-relativistic Douglas–Kroll (no-pair) spin-averaged approximation [29]. At the CCSD(T)/[13s11p7d4f/_Au_5s3p2d/_B_] computational level, they found values of *r*_e_ = 1.960 Å, *D*_e_ = 2.709 eV, *ω*_e_ = 663.0 cm^−1^, and *ω*_e_*x*_e_ = 4.01 cm^−1^. In 2010, via B3LYP/LANL2DZ and SSD, values of *D*_e_ = 2.96 eV and 3.04 eV, respectively, were calculated [48]. In 2020, Wang et al. investigated the nature of the chemical bonding in AuB, employing high-resolution photoelectron imaging and theoretical calculations [47]. They calculated r*_e_* = 1.906 Å using B2PLYP)/aug-cc-pVQZ(-PP) and a dissociation energy of 3.734 eV at the CCSD(T) level of theory [47]. This dissociation energy was in excellent agreement with the experimental value of 3.724(3) eV measured by Merriles and Morse in 2023 [30], who examined the AuB molecule experimentally using R2PI spectroscopy. They found that it remains bound at energies that far surpass its bond dissociation energy (BDE), and bonds break only when excited at or above an excited sharp predissociation threshold (SAL) [30].

**HgB:** There is only one theoretical study on HgB. The ground state was assigned to be the *Χ*^2^Σ^+^ via the B3LYP/LANL2DZ and B3LYP/SDD methodologies. Values of *r*_e_ = 4.381 (2.535) Å, *ω*_e_ = 19 (177) cm^−1^, *μ* = 0.27 (1.20) D, EA = 0.63 (0.90) eV, IP = 7.10 (7.40) eV, and D_e_ = 0.002(0.17) eV were calculated at the B3LYP/LANL2DZ(B3LYP/SDD) levels of theory [48]. The differences between the two methods indicate that more accurate calculations should be performed using larger basis sets than the double zeta quality of the LANL2DZ and SDD basis sets.

## 3. Results and Discussion

DFT calculations were carried out for the ground states of the ZnB and the second- and third-row-transition-metal monoborides, MBs, and for some low-lying excited states of the NbB, TcB, LaB, TaB, ReB, and HgB molecules. Three functionals were used, i.e., B3LYP [55], MN15 [56], and TPSSh [57], in conjunction with the aug-cc-pVQZ basis set for B and the aug-cc-pVQZ-PP basis set [58,59,60,61] for all M except La; for La, the def2-QZVPPD basis set was used [62]. Bond distances, dissociation energies with respect to the adiabatic products and ground state products, frequencies, dipole moments, and natural NPA charges were calculated; Table 4 and Table 5. In the case of the NbB molecule, additional MRCISD(+Q)/aug-cc-pVQZ(-PP) calculations were carried out to clarify its ground state. Finally, the chemical bonding was analyzed, and it is presented in Table 6.

### 3.1. Second-Row-Transition-Metal Borides

The least studied molecule is the TcB species, where only a calculated *D*_0_ value of 3.31 eV via the B97-1/(aug)-cc-pVTZ-(PP) methodology has been reported [25]. Here, we calculated three electronic states, *X*^3^Σ^−^, ^5^Σ^−^, and ^7^Σ^−^, and all the main spectroscopic data are provided; see Table 4. The ground state is a triplet state with a bond distance of 1.746 Å and a dissociation energy of 4.838 eV with respect to the adiabatic products Tc(^4^D)[4*d*^6^(^5^D)5*s*] + B(^3^P). At equilibrium, the Tc atom is excited at the ^4^F[4*d^7^*] state and it forms a quadruple bond, *σ*^2^*σ*^2^*π_x_*^2^*π_y_*^2^, with the boron atom; see Table 6. The dissociation energy of the ground state with the atomic ground state atoms is 3.894 eV; see discussion below. The lowest quintet and septet states are of Σ^−^ symmetry, i.e., ^5^Σ^−^, and ^7^Σ^−^, and they lie 0.312 eV and 2.512 eV above the ground state. They are adiabatically correlated with the Tc(^6^S[4*d*^5^5*s*^2^]) + B(^3^P) dissociation channel, but at equilibrium, the Tc atom is excited at the Tc(^6^D[4*d*^6^(^5^D)5*s*]) + B(^3^P) channel. Their dissociation energies with respect to the atomic ground state products are 3.582 eV and 1.382 eV, respectively. The corresponding bond distances are 1.829 Å and 2.080 Å and the formed bonds are *σ*^1^*π_x_*^2^*π_y_*^2^ and *σ*^1^*π*^1^, respectively. The decrease in the formed bonds from the triplet to the quintet and septet can be perceived in the corresponding decrease in the dissociation energy and the corresponding increase in the bond distance. The dissociation energy per bond is about 1.2 eV in all three states, i.e., 1.21 eV (*X*^3^Σ^−^), 1.02 eV (^5^Σ^−^), and 1.38 eV (^7^Σ^−^).

**Table 4:** Bond lengths *r*_e_ (Å), dissociation energies *D*_e_ (eV) with respect to the adiabatic atomic products (with respect to the ground state atomic products, in parenthesis), vibrational frequencies *ω*_e_ (cm^−1^), dipole moments *μ* (Debye), and charge on metal *q*_M_ via natural population analysis of ground and some low-lying states of the 2nd-row-transition-metal boride molecules, MBs (M = Y, Zr, Nb, Mo, Tc, Ru, Rh, Pd, Ag, and Cd) at the B3LYP, TPSSh, and MN15/aug-cc-pVQZ(-PP) levels of theory and for the NbB molecule using the MRCISD and MRCISD+Q/aug-cc-pVQZ(-PP) methodologies.

MB	State	*r*_e_	*D*_e_	*ω*_e_	*μ*	*q*_M_
			**B3LYP**			
**YB**	*X*^5^Σ^−^	2.231	3.261 (2.094)	571.9	4.894	+0.59
**ZrB**	*X*^6^Δ	2.162	3.035 (2.787)	604.2	3.550	+0.30
**NbB**	*X*^5^Π ^a^	1.988	2.862	692.5	3.025	+0.15
	*a*^3^Σ^+^	1.870	2.755 (2.460)	805.8	2.984	+0.18
**MoB**	*X*^6^Π	1.973	2.247	654.9	2.221	+0.00
**TcB**	*X*^3^Σ^−^	1.746	4.838 (3.894)	848.3	3.767	+0.00
	*a*^5^Σ^−^	1.829	3.582	796.3	1.785	−0.13
	^7^Σ^−^	2.080	1.382	516.0	2.240	+0.23
**RuB**	*X*^2^Δ	1.700	5.210 (4.463)	935.6	3.480	−0.09
**RhB**	*X*^1^Σ^+^	1.679	5.491 (4.969)	960.6	2.877	−0.18
**PdB**	*X*^2^Σ^+^	1.777	3.781	759.7	1.281	−0.26
**AgB**	*X*^1^Σ^+^	2.070	1.900	457.5	1.296	−0.11
**CdB**	*X*^2^Π	2.466	0.305	237.1	1.684	+0.24
			**TPSSh**			
**YB**	*X*^5^Σ^−^	2.235	3.418 (2.475)	574.1	4.893	+0.60
**ZrB**	*X*^6^Δ	2.166	3.247 (3.301)	608.4	3.691	+0.31
**NbB**	*X*^5^Π ^a^	1.992	3.010	690.6	3.229	+0.17
**MoB**	*X*^6^Π	1.982	2.338	646.0	2.416	+0.02
**TcB**	*X*^3^Σ^−^	1.765	5.210 (4.222)	835.7	3.766	+0.01
**RuB**	*X*^2^Δ	1.711	5.518 (4.443)	915.0	3.604	−0.07
**RhB**	*X*^1^Σ^+^	1.685	5.412 (4.991)	958.4	3.079	−0.16
**PdB**	*X*^2^Σ^+^	1.781	4.020	768.8	1.543	−0.24
**AgB**	*X*^1^Σ^+^	2.056	1.980	474.9	1.555	−0.10
**CdB**	*X*^2^Π	2.413	0.483	274.7	1.953	+0.26
			**MN15**			
**YB**	*X*^5^Σ^−^	2.210	3.649 (1.969)	590.9	5.095	+0.59
**ZrB**	*X*^6^Δ	2.137	3.347 (2.651)	624.7	3.670	+0.29
**NbB**	*X*^5^Π ^a^	1.968	3.056	718.3	3.006	+0.12
**MoB**	*X*^6^Π	1.942	2.465	709.0	1.991	−0.06
**TcB**	*X*^3^Σ^−^	1.724	5.390 (4.899)	915.1	3.589	−0.06
**RuB**	*X*^2^Δ	1.684	5.221 (5.169)	986.9	3.375	−0.14
**RhB**	*X*^1^Σ^+^	1.666	6.064 (5.895)	1002.6	2.850	−0.22
**PdB**	*X*^2^Σ^+^	1.762	4.056	783.4	1.084	−0.30
**AgB**	*X*^1^Σ^+^	2.053	2.151	470.5	1.155	−0.15
**CdB**	*X*^2^Π	2.462	0.366	218.5	1.238	+0.20
			**MRCISD**			
**NbB**	*X*^5^Π	2.017	2.799 ^b^	709.0	3.096	+0.16
	*A*^5^Φ	2.018	2.715 ^b^	710.4	2.921	+0.16
			MRCISD+Q			
	*X*^5^Π	2.018	2.901 ^b^	708.9		
	*A*^5^Φ	2.019	2.808 ^b^	710.3		

^a^ The *X*^5^Π and ^5^Φ are almost energetically degenerate. *A*^5^Φ lies 0.084(0.093) eV above the *X*^5^Π state at the MRCISD(MRCISD+Q) level of theory. ^b^ Dissociation energy obtained via potential energy cure at infinite r_e_ values.

Regarding the ground state of NbB, the *X* state correlates to the atomic ground state products Nb(^6^D[4*d*^4^5*s*^1^]) + B(^2^P) and it maintains this character in the minimum. DFT cannot predict if the state is a ^5^Π(1) or ^5^Φ(3), i.e., *m_ℓ_*(Nb) = *±*2, +*m_ℓ_*(B) = ±1, and thus multireference techniques are necessary to clarify the electronic state. MRCISD(+Q)/aug-cc-pVQZ(-PP) calculations were carried out and it was found that the global minimum is an *X*^5^Π state and the *A*^5^Φ lies 0.084(0.093) eV above the *X* state. Their *r*_e_ values were calculated to be 2.017(2.018) Å for the *X*^5^Π state and 2.018(2.019) Å for the *A*^5^Φ, respectively. The lowest triplet state is the *a*^3^Σ^+^, which lies 0.402 eV above the *X* state. The bonding in the *X* state is 12(σ1πx2πy1±σ1πx1πy2), while in the *a*^3^Σ^+^ it is *σ*^1^*π_x_*^2^*π_y_*^2^; see Table 6. The multiple bonding of 2 in the *X* state in contradiction to 2.5 in the ^3^Σ^+^ affects the corresponding bond distances, i.e., 1.988 vs 1.870, where a decrease of 0.1 Å is observed.

**Figure 1:**
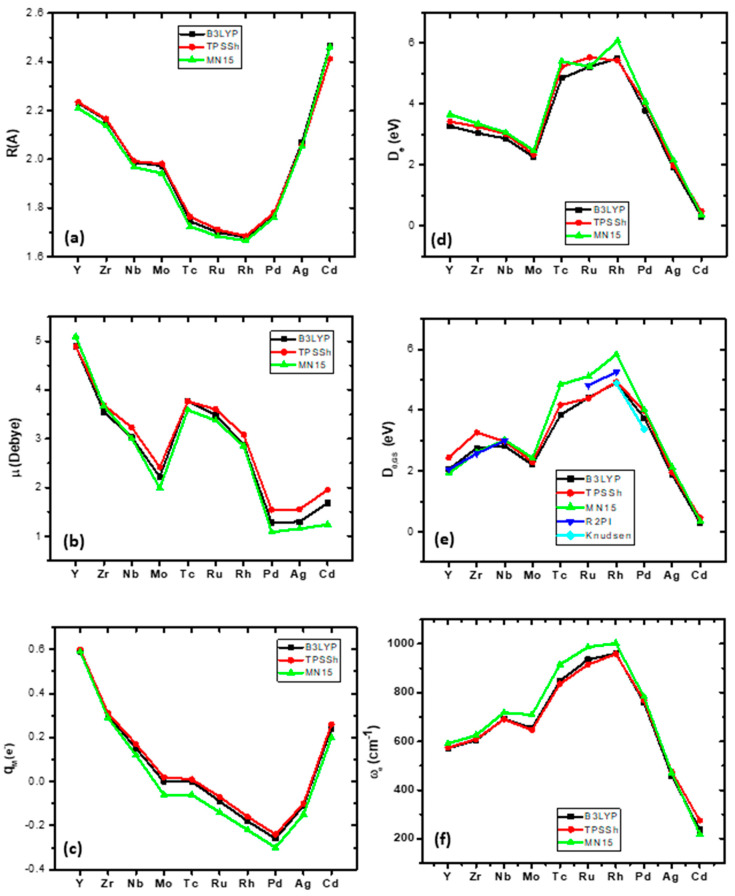
(**a**) Bond lengths, *r*_e_, (**b**) dipole moments, *μ*, (**c**) charge on metal, *q*_M_, via natural population analysis, (**d**) dissociation energies, *D*_e_ (eV), with respect to the adiabatic atomic products, (**e**) dissociation energies, *D*_0,GS_, with respect to the ground state atomic products, and (**f**) vibrational frequencies, *ω*_e_, of the ground states of the 2nd-row-transition-metal boride molecules, MBs (M = Y, Zr, Nb, Mo, Tc, Ru, Rh, Pd, Ag, and Cd), at the B3LYP, TPSSh, and MN15/aug-cc-pVQZ(-PP) level of theory.

The bond distances of the ground states of the second-row-transition-metal borides, MBs, with respect to the different M atoms, are plotted in Figure 1a. Note that all three functionals present the same geometry; see Table 4. YB has a bond distance of 2.231 Å, and as the M changes along the row of the periodic table, the bond distance decreases up to RhB (1.679 Å) and then increases up to CdB at 2.466 Å.

The dissociation energy with respect to the adiabatic products (*D*_e_) as the M atom changes from left to right is plotted in Figure 1d. The *D*_e_ value is decreased from YB up to MoB; then, the TcB, RuB, and RhB molecules have similar *D*_e_ values at ~5eV, i.e., 4.838 eV, 5.210 eV, and 5.491 eV, respectively; finally, the *D*_e_ values decrease constantly up to CdB at 0.305 eV. The dissociation energy with respect to the atomic ground state products at the zero-vibrational level, *D*_0,GS_, with respect to the change in the M atom from left to right, is plotted in Figure 1e, where we observe an increase in the *D*_0,GS_ value up to NbB, then a small decease for the MoB, and then an increase up to Rh. Furthermore, the available experimental *D*_0_ values determined via R2PI and Knudsen spectrometry are plotted in Figure 1e. The calculated *D*_0,GS_ values are in very good agreement with the experimental values; the % differences range from 0.00 to 0.09%.

The dipole moment, *μ*, follows a decreasing pattern from 5.10 Debye (YB) to 1.99 D (MoB), followed by an immediate jump at 3.59 D (TcB), and then by a small decrease at 3.38 D (RuB) and at 2.85 D (RhB). Note that a quadruple bond is formed in these three molecules. Finally, the last three MBs have similar dipole moments, i.e., 1.08 D (PdB), 1.16 D (AgB), and 1.24 D CdB; see Figure 1b.

The NPA charge on metal is positive in YB, +0.6*e*, i.e., about half of the electron has been moved to the B atom, and it decreases to 0 for MoB and TcB. In these two molecules, there is a charge transfer from M to B and back. Then, from Ru to Ag, the metal has a total negative charge, i.e., the charge is transferred from B to M, while in CdB, again the M is charged positively, which is expected since the M has an *s*^2^*d*^10^ atomic configuration (Figure 1c). The opposite trend is observed in harmonic vibrational frequencies, where the *ω*_e_ values increase from Y (571.9 cm^−1^) to Rh (960.6 cm^−1^) and then decreased to Cd (237.1 cm^−1^); see Figure 1f.

The bonding of the ground and excited states of the MBs is reported in Table 6. In the *X* states of the first two MB molecules, YB(*X*^5^Σ^−^) and ZrB(*X*^6^Δ), three half bonds, *σ*^1^*π*^1^*π*^1^, are formed. The additional electron in ZrB is added to the non-bonding *δ*(*d_x_*_²−*y*²_ or *d_xy_*) orbital. As a result, they have *r*_e_ values that differ only by about 0.07 Å, and their *D*_e_ values differ only by about 0.2 eV. The next NbB(*X*^5^Π) and MoB(*X*^6^Π) form two half bonds and one whole bond, *σ*^1^*π*^2^*π*^1^. The additional e^−^ from Zr to Nb is placed in the half-occupied *π* orbital, while the additional electron of MoB is added to the non-bonding *δ*(*d_x_*_²−*y*²_ or *d_xy_*) orbital. As a result, the *X* states of NbB and MoB have *r*_e_ values that differ only by about 0.01 Å and *D*_e_ values that differ by about 0.6 eV.

**Figure 2:**
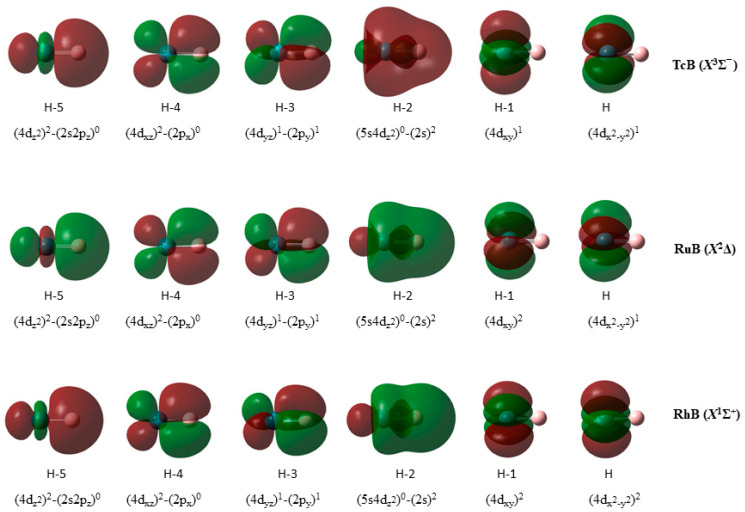
Molecular orbitals of the *X*^3^Σ^−^ (TcB), *X*^2^Δ (RuB), and *X*^1^Σ^+^ (RhB) states presenting quadruple bonds.

Then, in TcB(*X*^3^Σ^−^), RuB(*X*^2^Δ), and RhB(*X*^1^Σ^+^), there is a significant change in the bonding compared to the first four MBs of the second row. The bonding is formed from an atomic state which has an empty 5*s* orbital from, for example, Tc(^4^F[4*d*^7^]), Ru(*b*^3^F[4*d*^8^]), and Rh(*a*^2^D[4*d*^9^]). As a result, the 5*s* of the metal is empty and thus it can accept electrons, forming a dative bond. The bonding in *X* states is 1*σ*^2^2*σ*^2^1*π_x_*^2^1*π_y_*^2^; see Figure 2. Specifically, the bonding is 1*σ*^2 =^ (M4*d_z_*_²_)^2^*→*(B2*p_z_*)^0^, which is a dative bond from the M to the B atom; 2*σ*^2 =^ (M5*s*M4*d_z_*_²_)^0^*←*(B2*s*)^2^, also a dative bond from the B to the M; and 1*π_x_*^2^ = (M4*d_xz_*)^1^ − (B2*p_x_*)^1^ and 1*π_y_*^2^ = (M4*d_yz_*)^1^ − (B2*p_y_*)^1^, which are both *π* covalent bonds. The similar bonding leads to similar short *r*_e_ values, smaller up to 0.3 Å with respect to MoB, and dissociation energies of about 5 eV (4.84 up to 5.49 eV). Note that there is a 5*s*5*d_z_*_²_ hybridization in M and a 2*s*2*p_z_* hybridization in B. The added electrons from Tc to Rh are added to non-bonding *δ* orbitals, i.e., *δ*^1^*δ*^1^ in TcB, *δ*^2^*δ*^1^ in RuB, and *δ*^2^*δ*^2^ in RhB.

The PdB(*X*^2^Σ^+^) also has an *X* state that is formed from an atomic state with an empty 5*s* orbital in Pd(^1^S[4*d*^10^]). However, there is a triple bond which consists of three dative bonds, i.e., 1*σ*^2^ = (Μ5*s*)^0^*←*(Β2*s*)^2^, 1*π_x_*^2^ = (Μ4*d_xz_*)^2^*→*(Β2*p_x_*)^0^, and 1*π_y_*^2^ = (Μ4*d_yz_*)^2^*→*(Β2*p_y_*)^0^, while there is a non-bonding *σ*^1^ which corresponds to the (Β2*p_z_*)^1^ of B. The AgB(*X*^1^Σ^+^) molecule also has a triple bond with one covalent and two dative bonds, i.e., 1*σ*^2 =^ (M5*s*)^1^-(B2*p_z_*)^1^, 1*π_x_*^2^ = (M4*d_xz_*)^2^*→*(B2p_x_)^0^ and 1*π_y_*^2^ = (M4*d_yz_*)^2^*→*(B2*p_y_*)^0^. Finally, CdB(*X*^2^Π) has two dative bonds, i.e., 1*π_x_*^2^ = (M4*d_xz_*)^2^*→*(B2*p_x_*)^0^ and 1*π_y_*^2^ = (M4*d_yz_*)^2^*→*(B2*p_y_*)^0^, resulting in a positive charge of +0.24*e* on Cd.

### 3.2. Third-Row-Transition-Metal Borides

The ground states of the third-row MB molecules were calculated, while for the LaB, TaB, ReB, and HgB molecules, an additional electronic state was also included; see Table 5 and Table 6. The ground state of the LaB is the *Χ*^5^Σ^−^ state, which correlates to La(^4^F[5*d*^2^6*s*]) + B(^2^P) and maintains this character in the minimum. Its dissociation energy, *D*_0_, with respect to the ground state products is 2.086 eV, in excellent agreement with the R2PI experimental value of 2.086 eV [25]. On the contrary, the ^3^Π state, which is 0.448 eV above the *X* state, correlates to an excited La(^2^D[5*d*^2^6*s*]), but the in situ La atom is b^4^F[5*d*^3^]. The *X* state has a *σ*^1^*π_x_*^1^*π_y_*^1^ bonding scheme, while the ^3^Π state presents a *σ*^1^*π_x_*^2^*π_y_*^1^ bonding scheme i.e., one less bonding electron, and, as a result, a shorter bond distance by 0.1 Å. The *X* state of the HfB is a *Χ*^4^Σ^−^ state and it has three half bonds with a dissociation energy of 2.810 eV, in good agreement with experimental value of 2.593 eV [25]. Two states have been calculated for the TaB; the *X*^5^Δ state, which has a whole bond and two half bonds, and the ^3^Σ^+^ state, which lies 0.260 eV above the ground and presents two and a half bonds. Again, the addition of one electron in the bonding results in a decrease in the bond distance by 0.1 Å. The dissociation energy of *X*^5^Δ with respect to the adiabatic products is 3.495 eV. The *X* state of WB is the *Χ*^6^Π, where a *σ*^1^*π*^2^*π*^1^ bond scheme is formed. It presents a dissociation energy of 2.907 eV and *r*_e_ = 1.955 Å. The ^6^Σ^+^ lies 0.137 eV above the X state; it forms a *σ*^2^*π_x_*^1^*π_y_*^1^ bond with a dissociation energy of 2.770 eV and *r*_e_ = 2.125 Å. The ground state of ReB is the *Χ*^5^Σ^−^, state which correlates to Re(^6^S[5*d*^5^6*s*^2^]), but the in situ atom is the Re(^6^D[5*d*^6^6*s*^1^]), which forms two and a half bonds. The dissociation energy is 3.106 eV and the bond distance at 1.834 Å. The *a*^3^Σ^−^ state lies only 0.099 eV above the ground state, and it has a stronger bonding, i.e., three bonds (*σ*^2^*π_x_*^2^*π_y_*^2^). Its bond distance is 1.809 Å, and its dissociation energy is 4.967 eV with respect to the correlated products, while with respect to the atomic ground state products, it is 3.008 eV.

**Table 5:** Bond lengths, *r*_e_ (Å); dissociation energies, *D*_e_ (eV) with respect to the adiabatic atomic products (with respect to the ground state atomic products, in parenthesis); vibrational frequencies, *ω*_e_ (cm^−1^); dipole moments, *μ* (Debye); and charge on metal *q*_M_ via natural population analysis of ground and some low-lying states of the 3rd-row-transition-metal boride molecules, MBs (M = La, Hf, Ta, W, Re, Os, Ir, Pt, Au, and Hg): at the B3LYP, TPSSh, and MN15/aug-cc-pVQZ_B_(-PP)_M_ and def2-QZVPPD_La_ levels of theory.

Molecule	State	*r*_e_ (Å)	*D*_e_	*ω*_e_	*μ*	*q*_M_
			**B3LYP**			
**LaB**	*Χ*^5^Σ^−^	2.384	2.874(2.528)	512.7	4.03	+0.55
	*a*^3^Π	2.263	2.080	478.5	6.08	+0.74
**HfB**	*Χ*^4^Σ^−^	2.151	2.810	594.8	2.55	+0.35
**TaB**	*X*^5^Δ	1.974	3.495	739.6	3.58	+0.22
	*a*^3^Σ^+^	1.870	3.236	838.2	3.78	+0.28
**WB**	*Χ*^6^ Π	1.955	2.907 ^a^	735.0	2.63	+0.04
	^6^Σ^+^	2.125	2.770 ^a^	548.2	2.86	+0.10
**ReB**	*Χ*^5^Σ^−^	1.834	3.106	865.5	2.28	−0.07
	*a*^3^Σ^−^	1.809	4.967(3.008)	850.7	2.85	−0.05
**OsB**	*Χ*^4^Σ^−^	1.772	4.482	935.1	2.18	−0.13
**IrB**	*Χ*^3^Δ	1.762	5.338	926.9	1.62	−0.24
**PtB**	*Χ*^2^Σ^+^	1.759	5.377	905.2	1.08	−0.32
**AuB**	*Χ*^1^Σ^+^	1.925	3.451	642.2	0.87	−0.26
**HgB**	*X*^2^Π	2.397	0.237	223.4	1.38	+0.17
**HgB**	*A*^2^Σ^+^	4.029	0.003	31.7	0.35	0.00
			**TPSSh**			
**LaB**	*Χ*^5^Σ^−^	2.384	3.032(2.786)	518.6	4.40	+0.56
**HfB**	*Χ*^4^Σ^−^	2.149	3.004	606.1	2.55	+0.36
**TaB**	*X*^5^Δ	1.979	3.585	733.5	3.65	+0.23
**WB**	*Χ*^6^ Π	1.963	2.938 ^a^	720.5	2.74	+0.06
	^6^Σ^+^	2.126	2.731 ^a^	549.5	2.99	+0.12
**ReB**	*Χ*^5^Σ^−^	1.838	3.519	863.3	2.41	−0.05
**OsB**	*Χ*^4^Σ^−^	1.779	4.646	909.9	2.59	−0.07
**IrB**	*Χ*^3^Δ	1.765	5.300	928.1	1.81	−0.22
**PtB**	*Χ*^2^Σ^+^	1.759	5.547	917.4	1.19	−0.31
**AuB**	*Χ*^1^Σ^+^	1.921	3.604	657.6	1.04	−0.25
**HgB**	*X*^2^Π	2.341	0.395	266.5	1.62	+0.19
**HgB**	*A*^2^Σ^+^	3.658	0.016	42.9	0.50	−0.009
			**MN15**			
**LaB**	*Χ*^5^Σ^−^	2.365	3.237(2.402)	526.1	4.64	+0.55
**HfB**	*Χ*^4^Σ^−^	2.133	3.123	603.5	2.46	+0.33
**TaB**	*X*^5^Δ	1.957	3.560	767.0	3.42	+0.19
**WB**	*Χ*^6^ Π	1.932	3.031 ^a^	778.1	2.47	0.00
**ReB**	*Χ*^5^Σ^−^	1.814	3.346	904.4	2.02	−0.11
**OsB**	*Χ*^4^Σ^−^	1.758	4.943	958.7	1.98	−0.17
**IrB**	*Χ*^3^Δ	1.750	5.633	944.7	1.46	−0.27
**PtB**	*Χ*^2^Σ^+^	1.744	5.744	934.8	0.87	−0.35
**AuB**	*Χ*^1^Σ^+^	1.902	3.933	673.9	0.69	−0.30
**HgB**	*X*^2^Π	2.337	0.364	234.4	1.12	+0.14
**HgB**	*A*^2^Σ^+^	3.388	0.085	66.9	0.68	−0.02

^a^ With respect to the W(^7^S) state, which is the lowest atomic state with respect to the average J term.

The OsB(*Χ*^4^Σ^−^), IrB(*Χ*^3^Δ), and PtB(*Χ*^2^Σ^+^) molecules present triple bonds in their ground states, with similar bond distances of 1.772 Å, 1.762 Å, and 1.759 Å, which is expected due to the same bonding. Note that the additional electron moving from Os to Pt is added to the non-bonding *d*_+2_ or *d*_−2_ atomic orbital. The dissociation energies with respect to the adiabatic products are 4.482 eV for OsB, 5.338 eV for IrB, and 5.377 eV for PtB. The IrB and OsB molecules correlate to their in situ atoms, while for OsB, the state of the M atom in the minimum differs from the correlated one. Thus, the dissociation energy of OsB with respect to its in situ atoms is 5.12 eV, only 0.2 eV less than the *D*_e_ value of IrB and PtB. On the contrary, whilst a triple bond is also formed in the *X* state of AuB (*Χ*^1^Σ^+^), the two *π*^2^ bonds which are dative, i.e., Au(5*d_xz_*)^2^*→*B(2*p_x_*)^2^ and Au(5*d_yz_*)^2^*→*B(2*p_y_*)^0^, are weak with a small charge transfer. Finally, two states were calculated for the HgB molecule, *X*^2^Π and *A*^2^Σ^+^. The *X* state has a weak double bond with an elongated bond distance of 2.397 Å and a dissociation energy of 0.237 eV, while the *A*^2^Σ^+^ is a van der Waals state, where very weak interactions are formed between atoms. Comparing the *D*_0_ dissociation energies (with respect to the ground state products) with the corresponding experimental values, there is a very good agreement, i.e., calculated[expt] values of 4.424[4.378] eV for OsB, 5.281[4.928] eV for IrB, 5.321[5.235] eV for PtB, and 3.411[3.724] eV for AuB. Thus, differences in D_0_ that range from 0.05 eV to 0.35 eV are observed. 

**Figure 3:**
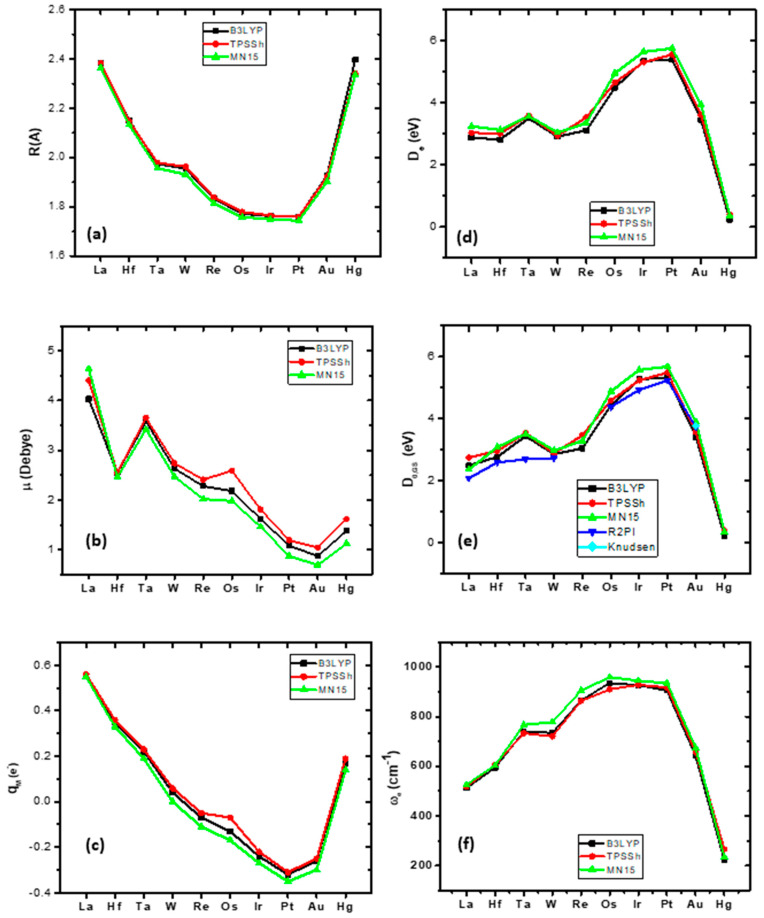
(**a**) Bond lengths, *r*_e_, (**b**) dipole moments, *μ*, (**c**) charge on metal, *q*_M_, via natural population analysis, (**d**) dissociation energies, *D*_e_ (eV) with respect to the adiabatic atomic products, (**e**) dissociation energies, *D*_0,GS_, with respect to the ground state atomic products, and (**f**) vibrational frequencies, *ω*_e_, of the ground states of the 3rd-row-transition-metal boride molecules, MBs (M = La, Hf, Ta, W, Re, Os, Ir, Pt, Au, and Hg) at the B3LYP, TPSSh, and MN15/aug-cc-pVQZ_B_(-PP)_M_ and def2-QZVPPD_La_ levels of theory.

The bond distances of the ground states of the third-row-transition-metal MB molecules, with respect to the different M atoms, are plotted in Figure 3a. Note that all three functionals present the same geometry (Table 5). The first molecule of the row, LaB, has a bond distance of 2.384 Å, and, as the M changes along the row of the periodic table, the bond distance decreases up to PtB (1.759 Å) and then increases up to HgB at 2.397 Å. It should be noted that OsB(*Χ*^4^Σ^−^), IrB(*Χ*^3^Δ), and PtB(*Χ*^2^Σ^+^) present similar bond distances, i.e., 1.772 Å, 1.762 Å, and 1.759 Å, respectively, and similar triple bonding, *σ*^2^*π*^2^*π*^2^.

The dipole moment, *μ*, follows a decreasing pattern from 4.03 Debye (LaB) to 0.87 D (AuB), except for the increase from Hf to Ta. Finally, there is an increase from AuB to HgB at 1.38 D; see Figure 3b. The NPA charge on the metal is positive in LaB, +0.55*e*, i.e., about half of the electrons are moved to the B atom, and the charge decreases to WB (+0.04 *e*); see Figure 3c. Then, from Re to Au, the metal has a total negative charge, i.e., the charge is transferred from B to M, while in HgB, again, the M is charged positively (+0.17*e*), which is expected since the M has an *s*^2^*d*^2^ atomic configuration. In PtB, the Pt demonstrates the most negative charge of all the metals. Finally, the harmonic vibrational frequencies generally increase from La to OsB. OsB, IrB, and PtB have similar *ω*_e_ values at about ~920 cm^−1^; then, they decrease to HgB (223.4 cm^−1^); see Figure 3f.

The dissociation energy with respect to the adiabatic products, *D*_e_, as the M atom changes from left to right is plotted in Figure 3d. The first five MBs have similar *D*_e_ values, with some discrepancies. Then, in OsB, IrB, and PtB, the *D*_e_ value increases; their values are 4.482 eV, 5.338 eV, and 5.377 eV, respectively, and finally, the *D*_e_ values decrease constantly up to HgB at 0.237 eV. The dissociation energy with respect to the atomic ground state products at the zero-vibrational level, *D*_0,GS_, with respect to the change in the M atom is plotted in Figure 3e. The available experimental *D*_0_ values are also plotted in Figure 3e via R2PI and Knudsen spectrometry. The calculated *D*_0,GS_ values for HfB, OsB, WB, and PtB are in excellent agreement, and for LaB and IrB, they are in good agreement, while the largest difference is observed for TaB. Finally, it should be noted that the B3LYP-obtained values are in better agreement with the experimental ones than the values obtained via the MN15 and TPSSh functional methods.

The bonding of the ground and excited states of the MBs is reported in Table 6. In the ground states of the first two MB molecules, i.e., LaB and HfB, three half bonds, *σ*^1^*π_x_*^1^*π_y_*^1^, are formed. As a result, they have the same dissociation energies, i.e., 2.874 eV and 2.810 eV. It should be noted that there is a strong 5*d_z_*_²_6*s* hybridization in the M atom. The TaB and WB form two half bonds and one whole bond, i.e., *σ*^1^*π_x_*^2^*π_y_*^1^. The following ReB has one half and two bonds, i.e., *σ*^1^*π_x_*^1^*π_y_*^2^, while the next four molecules, OsB, IrB, PtB, and AuB, form a triple bond, i.e., *σ*^2^*π_x_*^2^*π_y_*^2^. Note that, contrary to the second row, where the atomic states of M atoms with empty 5*s* orbitals are involved in the bond, the corresponding atomic states of the M atoms occupy the 6*s* atomic orbital. Finally, the last MB of this row, i.e., HgB, has two dative bonds, i.e., 1*π_x_*^2^ = M(5*d_xz_*)^2^*→*B(2*p_x_*)^0^ and 1*π_y_*^2^ = M(5*d_yz_*)^2^*→*B(2*p_y_*)^0^, similar to the CdB of the second row, thus leading to similar bond lengths, 2.466 Å (CdB) and 2.397 Å (HgB), and dissociation energies, 0.305 eV and 0.237 eV, respectively. 

### 3.3. Comparison of MBs of All Three Rows and Bonding Analysis

Quantum chemical computations provide details on the chemical bonds and electronic structures of these species. In general, the bond lengths of the transition-metal borides increase in the periodic table from left to right, or as one goes down a group of elements. Of course, anomalies occur, but the data are the result of the variety of bonding schemes that are formed in the MB molecules. These bonding schemes depend on the bonding and the filling of *σ*, *π*, and *δ* orbitals. MBs can form one-and-a-half, double, triple, and even quadruple bonds, with the latter being recently discovered in RhB and RhB^−^ [2,4,5], while here, we found that RuB and TcB also form quadruple bonds.

**Figure 4:**
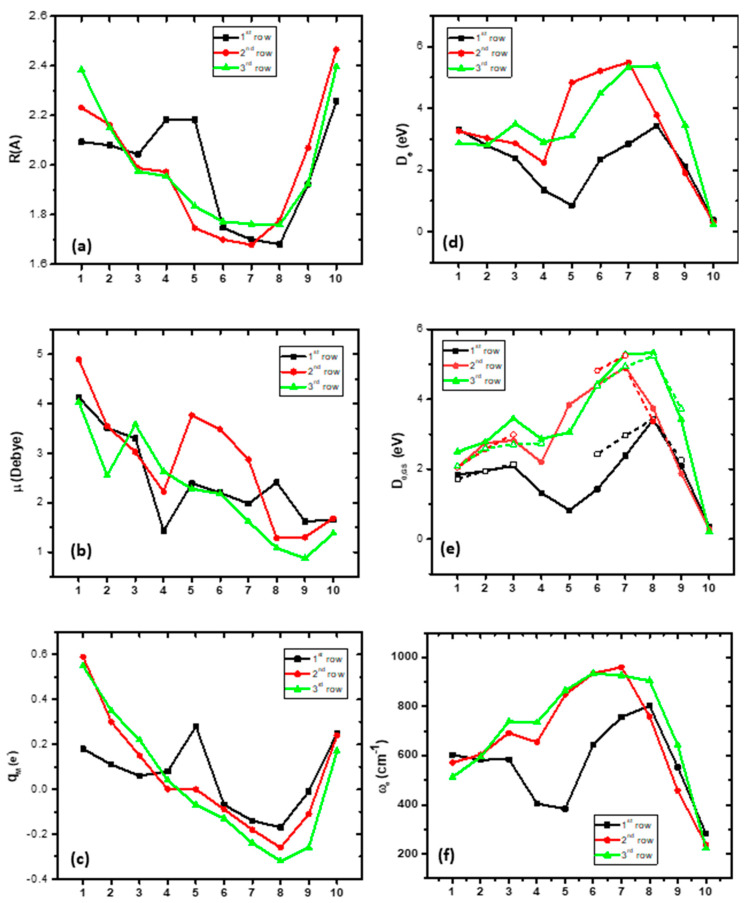
(**a**) Bond lengths, *r*_e_, (**b**) dipole moments, *μ*, (**c**) charge on metal, *q*_M_, via natural population analysis, (**d**) dissociation energies, *D*_e_ (eV), with respect to the adiabatic atomic products, (**e**) dissociation energies, *D*_0,GS_, with respect to the ground state atomic products, and (**f**) vibrational frequencies, *ω*_e_, of the ground states of the 1st-, 2nd-, and 3rd-row-transition-metal boride molecules; 1st row: MRCISD+Q/aug-cc-pV5Z [21]; 2nd- and 3rd-row MBs: B3LYP/aug-cc-pVQZ_B_(-PP)_M_ and def2-QZVPPD_La_ levels of theory.

The bond distances of the ground states of the MBs of all three rows, with respect to the different M atoms, are plotted in Figure 4a. Moving from up (first row) to down (third row) the bond distances, in the cases where there is the same bonding, the *r*_e_ values increase by about 1 Å. Also, moving from left to right, the *r*_e_ value decreases up to the seventh or eighth MB, and then the *r*_e_ value increases sharply. All differences are a result of the type of bonding. In all rows, there are three MBs that present similar strong bonding. Thus, along the first row, from the sixth to the eight MB, i.e., FeB, CoB, and NiB, a triple bond and similar *r*_e_ values, around 1.7 Å, are observed. Similarly, along the third row, from the sixth to the eighth MB, OsB, IrB, and PtB, a triple bond and similar *r*_e_ values, around 1.76 Å, are found. On the contrary, in the second row, from the fifth to the seventh MB, i.e., TcB, RuB, and RhB, a quadruple bond is formed with similar *r*_e_ values, around 1.7 Å.

In Figure 4d,e, the dissociation energies with respect to the adiabatic products (*D*_e_) and the atomic ground state products at the zero-vibrational level (*D*_0,GS_) are plotted with respect to the change in the M atom from left to right for the three rows. The general shape is the same as that of the lowest dissociation energies being observed for the M, with atomic configurations of (*ds*)^6^ or (*ds*)^7^ and *d*^10^*s*^2^, while the highest values are observed for the M with atomic configurations of (*ds*)^8–10^ for the first and third rows and (*ds*)^7–9^ for the second row, and the largest values are found for the second and third rows. Finally, it should be noted that the first two and the last two MBs present similar dissociation energy regardless of the row.

The dipole moment, *μ*, with respect to the M follows roughly the same trend as the M changes from left to right in each row, i.e., a general reduction in the *μ* value. Any differences are related to the differences in the bonding; see Figure 4b. The smallest differences among the three rows are observed in *d*^1^*s*^2^, (*ds*)^5^, and *d*^10^*s*^2^, while the first and third rows present similar *μ* values for the *d*^5^*s*^2^ metals, where the second-row MB presents a *μ* value larger by 1.5 D than the corresponding MnB and ReB values due to the quadruple bond of TcB. 

**Table 6:** Atomic states of transition metals forming the bonding (in situ M) of the ground MB state (*X*) and the atomic state of M in *R*_M-B_ infinity; and of the atomic ground state *X*_M_ and the energy difference between the ground atomic state and the atomic state forming the bonding *T*_e_ (eV) (*M_J_*-averaged experimental).

MB	X	Configuration	Bond	In Situ M	In R_M-B_ Infinity	X_M_	Τ_e_ ^b^
		**1st row**		**MB**		**M**	
**ScB**	**X^5^Σ** ^−^	1σ^2^2σ^1^3σ^1^1π^1^1π^1^	σ^1^π_x_^1^π_y_^1^	^4^F[3d^2^4s^1^] ^a^	^4^F[3d^2^4s^1^]	^2^D[3d^1^4s^2^]	1.428(1.427)
**TiB**	***X*** **^6^** **Δ**	1σ^2^2σ^1^3σ^1^1π^1^1π^1^1δ^1^	σ^1^π_x_^1^π_y_^1^	^5^F[3d^3^(^4^F)4s^1^] ^a^	^5^F[3d^3^(^4^F)4s^1^]	a^3^F[3d^2^4s^2^ ]	0.813(0.806)
**VB**	***X*** **^7^** **Σ** ^+^	1σ^2^2σ^1^3σ^1^1π^1^1π^1^1δ^1^1δ^1^	σ^1^π_x_^1^π_y_^1^	^6^D[3d^4^(^5^D)4s^1^] ^a^	^6^D[3d^4^(^5^D)4s^1^]	a^4^F[3d^3^4s^2^]	0.262(0.245)
**CrB**	***X*** **^6^** **Σ** ^+^	1σ^2^2σ^2^3σ^1^1π^1^1π^1^1δ^1^1δ^1^	σ^2^π_x_^1^π_y_^1^	^7^S[3d^5^(^6^S)4s^1^] ^a^	^7^S[3d^5^(^6^S)4s^1^]	^7^S[3d^5^(^6^S)4s^1^]	0
**MnB**	***X*** **^5^** **Π**	1σ^2^2σ^2^3σ^1^1π^2^1π^1^1δ^1^1δ^1^	σ^2^π_x_^2^π_y_^1^	^6^S[3d^5^4s^2^] ^a^	^6^S[3d^5^4s^2^]	a^6^S[3d^5^4s^2^]	0
**FeB**	**Χ^4^Σ^−^**	1σ^2^2σ^2^3σ^1^1π^2^1π^2^1δ^1^1δ^1^	σ^2^π_x_^2^π_y_^2^	a^5^F[3d^7^(^4^F)4s^1^] ^a^	a^5^D[3d^6^4s^2^]	a^5^D[3d^6^4s^2^]	0.859(0.875)
**CoB**	**Χ^3^Δ**	1σ^2^2σ^2^3σ^1^1π^2^1π^2^1δ^2^1δ^1^	σ^2^π_x_^2^π_y_^2^	b^4^F[3d^8^(^3^F)4s^1^] ^a^	a^4^F[3d^7^4s^2^]	a^4^F[3d^7^4s^2^]	0.432(0.417)
**NiB**	**Χ^2^Σ** ^+^	1σ^2^2σ^2^3σ^1^1π^2^1π^2^1δ^2^1δ^2^	σ^2^π_x_^2^π_y_^2^	a^3^D[3d^9^(^2^D)4s^1^] ^a^	a^3^F[3d^8^(^3^F)4s^2^]	a^3^F[3d^8^(^3^F)4s^2^]	0.025(-0.030)
**CuB**	**Χ^1^Σ** ^+^	1σ^2^2σ^2^3σ^2^1π^2^1π^2^1δ^2^1δ^2^	σ^2^π_x_^2^π_y_^2^	^2^S[3d^10^(^1^S)4s^1^] ^a^	^2^S[3d^10^(^1^S)4s^1^]	^2^S[3d^10^(^1^S)4s^1^]	0
		**2nd row**					
**ZnB**	**Χ^2^Π**	1σ^2^2σ^2^3σ^2^1π^2^1π^2^2π^1^1δ^2^1δ^2^	σ^2^π^2^	^1^S[3d^10^4s^2^]	^1^S[3d^10^4s^2^]	^1^S[3d^10^4s^2^]	0
**YB**	**X^5^Σ** ^−^	1σ^2^2σ^1^3σ^1^1π^1^1π^1^	σ^1^π_x_^1^π_y_^1^	^4^F[4d^2^5s^1^]	a^4^F[4d^2^(^3^F)5s]	a^2^D[4d5s^2^]	1.356(1.359)
**ZrB**	***X*** **^6^** **Δ**	1σ^2^2σ^1^3σ^1^1π^1^1π^1^1δ^1^	σ^1^π_x_^1^π_y_^1^	^5^F[4d^3^(^4^F)5s^1^]	a^5^F[4d^3^(^4^F)5s^1^]	a^3^F[4d^2^5s^2^]	0.604(0.588)
**NbB**	**^5^Π**/**^5^Φ**	1σ^2^2σ^1^3σ^1^1π^2^1π^1^1δ^1^	σ^1^π_x_^2^π_y_^1^	a^6^D[4d^4^(^5^D)5s^1^]	a^6^D[4d^4^(^5^D)5s^1^]	a^6^D[4d^4^(^5^D)5s^1^]	0
	**^3^** **Σ** ^+^	1σ^2^2σ^1^3σ^1^1π^2^1π^2^	σ^1^π_x_^2^π_y_^2^	a^4^D[4d^4^5s^1^]	a^4^F[4d^3^5s^3^]	a^6^D[4d^4^(^5^D)5s^1^]	1.043(1.049)
**MoB**	**X^6^Π**	1σ^2^2σ^1^3σ^1^1π^2^1π^1^1δ^1^1δ^1^	σ^1^π_x_^2^π_y_^1^	a^7^S[4d^5^(^6^S)5s]	a^7^S[4d^5^(^6^S)5s]	a^7^S[4d^5^(^6^S)5s]	0
**TcB**	**X^3^Σ** ^−^	1σ^2^2σ^2^1π^2^1π^2^1δ^1^1δ^1^	σ^2^σ^2^π_x_^2^π_y_^2^	^4^F[4d^7^]	^4^D[4d^6^(^5^D)5s]	^6^S[4d^5^5s^2^]	1.827(2.332)
	**^5^** **Σ** ^−^	1σ^2^2σ^1^3σ^1^1π^2^1π^2^1δ^1^1δ^1^	σ^1^π_x_^2^π_y_^2^	^6^D[4d^6^5s^1^]	^6^S[4d^5^5s^2^]	^6^S[4d^5^5s^2^]	0.319(0.406)
	**^7^** **Σ** ^−^	1σ^2^2σ^1^3σ^1^1π^2^1π^1^2π^1^1δ^1^1δ^1^	σ^1^π^1^	^6^D[4d^6^5s^1^]	^6^S[4d^5^5s^2^]	^6^S[4d^5^5s^2^]	0.319(0.406)
**RuB**	**X^2^Δ**	1σ^2^2σ^2^1π^2^1π^2^1δ^2^1δ^1^	σ^2^σ^2^π_x_^2^π_y_^2^	b^3^F[4d^8^]	a^3^F[4d^7^(a^4^F)5s]	a^5^F[4d^7^(a^4^F)5s]	1.131(1.092)
**RhB**	**X^1^Σ** ^+^	1σ^2^2σ^2^1π^2^1π^2^1δ^2^1δ^2^	σ^2^σ^2^π_x_^2^π_y_^2^	a^2^D[4d^9^]	a^2^D[4d^9^]	a^4^F[4d^8^(^3^F)5s]	0.410(0.342)
**PdB**	**X^2^Σ** ^+^	1σ^2^2σ^2^3σ^1^1π^2^1π^2^1δ^2^1δ^2^	σ^2^π_x_^2^π_y_^2^	^1^S[4d^10^]	^1^S[4d^10^]	^1^S[4d^10^]	0
**AgB**	**X^1^Σ** ^+^	1σ^2^2σ^2^3σ^2^1π^2^1π^2^1δ^2^1δ^2^	σ^2^π_x_^2^π_y_^2^	^2^S[4d^10^5s]	^2^S[4d^10^5s]	^2^S[4d^10^5s]	0
**CdB**	**X^2^Π**	1σ^2^2σ^2^3σ^2^1π^2^1π^2^2π^1^1δ^2^1δ^2^	σ^2^π^2^	^1^S[4d^10^5s^2^]	^1^S[4d^10^5s^2^]	^1^S[4d^10^5s^2^]	0
		**3rd row**					
**LaB**	**X^5^Σ** ^−^	1σ^2^2σ^1^3σ^1^1π^1^1π^1^	σ^1^π_x_^1^π_y_^1^	^4^F[5d^2^(^3^F)6s]	^4^F[5d^2^(^3^F)6s]	^2^D[5d6s^2^]	0.331(0.355)
	**^3^** **Π**	1σ^2^2σ^1^1π^2^1π^1^	σ^1^π_x_^2^π_y_^1^	b^4^F[5d^3^]	^2^D[5d6s^2^]	^2^D[5d6s^2^]	1.541(1.560)
**HfB**	**X^4^Σ** ^−^	1σ^2^2σ^2^3σ^1^1π^1^1π^1^	σ^1^π_x_^1^π_y_^1^	a^3^F[5d^2^6s^2^]	a^3^F[5d^2^6s^2^]	a^3^F[5d^2^6s^2^]	0
**TaB**	**X^5^Δ**	1σ^2^2σ^1^3σ^1^1π^2^1π^1^1δ^1^	σ^1^π_x_^2^π_y_^1^	a^6^D[5d^4^6s^1^]	a^4^F[5d^3^6s^2^]	a^4^F[5d^3^6s^2^]	1.210(1.038)
	**^3^** **Σ** ^+^	1σ^2^2σ^1^3σ^1^1π^2^1π^2^	σ^1^π_x_^2^π_y_^2^		a^4^F[5d^3^6s^2^]	a^4^F[5d^3^6s^2^]	
**WB**	**X^6^Π**	1σ^2^2σ^1^3σ^1^1π^2^1π^1^1δ^1^1δ^1^	σ^1^π_x_^2^π_y_^1^	^7^S[5d^5^6s^1^]	^5^D[5d^4^6s^2^]	^5^D[5d^4^6s^2^]	0.366(−0.187)
	**^6^** **Σ** ^+^	1σ^2^2σ^2^3σ^1^1π^1^1π^1^1δ^1^1δ^1^	σ^2^π_x_^1^π_y_^1^	^7^S[5d^5^6s^1^]	^5^D[5d^4^6s^2^]	^5^D[5d^4^6s^2^]	0.366(−0.187)
**ReB**	**X^5^Σ** ^−^	1σ^2^2σ^1^3σ^1^1π^2^1π^2^1δ^1^1δ^1^	σ^1^π_x_^2^π_y_^2^	a^6^D[5d^6^6s^1^]	a^6^S[5d^5^6s^2^]	a^6^S[5d^5^6s^2^]	1.457(1.759)
	**^3^** **Σ** ^−^	1σ^2^2σ^2^1π^2^1π^2^1δ^1^1δ^1^	σ^2^π_x_^2^π_y_^2^	a^4^P[5d^5^6s^2^]	a^4^P[5d^5^6s^2^]	a^6^S[5d^5^6s^2^]	1.436(1.603)
**OsB**	**Χ^4^Σ** ^−^	1σ^2^2σ^2^3σ^1^1π^2^1π^2^1δ^1^1δ^1^	σ^2^π_x_^2^π_y_^2^	a^5^F[5d^7^(^4^F)6s^1^]	a^5^D[5d^6^6s^2^]	a^5^D[5d^6^6s^2^]	0.638(0.757)
**IrB**	**Χ^3^Δ**	1σ^2^2σ^2^3σ^1^1π^2^1π^2^1δ^2^1δ^1^	σ^2^π_x_^2^π_y_^2^	a^4^F[5d^7^6s^2^]	a^4^F[5d^7^6s^2^]	a^4^F[5d^7^6s^2^]	0
**PtB**	**Χ^2^Σ** ^+^	1σ^2^2σ^2^3σ^1^1π^2^1π^2^1δ^2^1δ^2^	σ^2^π_x_^2^π_y_^2^	^3^D[5d^9^6s^1^]	^3^D[5d^9^6s^1^]	^3^D[5d^9^6s^1^]	0
**AuB**	**Χ^1^Σ** ^+^	1σ^2^2σ^2^3σ^2^1π^2^1π^2^1δ^2^1δ^2^	σ^2^π_x_^2^π_y_^2^	^2^S[5d^10^6s^1^]	^2^S[5d^10^6s^1^]	^2^S[5d^10^6s^1^]	0
**HgB**	**Χ^2^Π**	1σ^2^2σ^2^3σ^2^1π^2^1π^2^2π^1^1δ^2^1δ^2^	σ^2^π^2^	^1^S[5d^10^6s^2^]	^1^S[5d^10^6s^2^]	^1^S[5d^10^6s^2^]	0
	**Χ^2^Σ** ^+^	1σ^2^2σ^2^3σ^2^4σ^1^1π^2^1π^2^1δ^2^1δ^2^	(π^2^π^2^)	^1^S[5d^10^6s^2^]	^1^S[5d^10^6s^2^]	^1^S[5d^10^6s^2^]	0

^a^ Ref. [21]. ^b^ Expt values of the energy separation between the term with the lowest in energy spin–orbit coupling angular momentum quantum number *J* (average term).

On the contrary, the NPA charge on the metals as the M changes from left to right has the same shape for all three rows apart, from the MnB of the first row; see Figure 4c. Finally, the change in the vibrational frequencies with respect to M for the three rows has the same trend; see Figure 4f. In most MB molecules, the second- and third-row MBs have larger *ω*_e_ values than the MBs of the first row.

**Figure 5:**
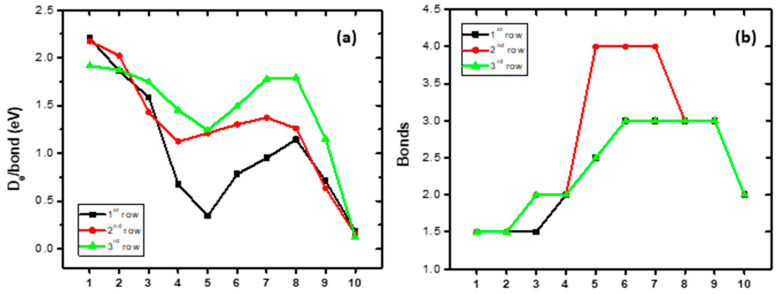
(**a**) Dissociation energies, *D*_e_ (eV), per bond with respect to the adiabatic atomic products; (**b**) number of formed bonds of the ground states of the 1st-, 2nd-, and 3rd-row-transition-metal boride molecules, MBs.

The dissociation energy per bond, i.e., per two bonding electrons, and the number of the formed bonds for all MBs of the first, second, and third rows are depicted in Figure 5. Regarding the diagram of the *D*_e_/bond, the general shape is the same for all three rows. The largest *D*_e_/bond, except for the first two and the last MBs, is observed for the MBs of the third row. Thus, the fact that the 4*d* series exhibits greater intrinsic bond energies [26] is explained with the formation of more multiple bonds than in some MBs of the 3*d* and 5*d* series, while the *D*_e_/bond is the highest for the third row, as expected.

Regarding the number of formed bonds, it seems that the MB molecules of the first and third rows present the same multiple bonds, except the (*n*)*d*^3^(*n* + 1)*s*^2^ atoms, where, for VB, the ground state is *X*^7^Σ^+^ and for ΤaB it is *X*^5^Δ. The difference in spin multiplicities results in different orders of bonding. The largest observed difference between MBs of the second row with the corresponding MBs of the first and third rows is for the fifth MB, i.e., TcB (quadruple bond) vs MnB and ReB (two and a half bonds). Finally, the X states of the RuB and RhB molecules of the second row also have quadruple bonds, while the corresponding MB molecules of the other rows have triple bonds.

The formation of quadruple bonding in the MB part of the complexes has been reported in the literature. For instance, in the anionic complex FeB(CO)_3_^−^, a quadruple bond is formed in the FeB part. The BFe(CO)_3_^−^ anion was calculated via DFT and DLPNO-CCSD(T) methodologies and identified using mass-selected infrared photodissociation spectroscopy in the gas phase [63].

Finally, it should be noted that in the transition-metal molecules, highly correlated electrons are involved in the spin−orbit interactions, and relativistic effects exist. The use of basis sets with pseudopotentials of course simplified the complicated effects of the motion of the core (non-valence) electrons. Furthermore, 5*d* MB molecules suffer from strong spin−orbit effects, which here have been neglected. The use of atomic spin−orbit stabilization [64] may lead to reductions in the calculated dissociation energies [25]. However, our calculated values are in very good agreement with the experimental ones, with the exception of TaB, since both M and MB species are subject to the spin–orbit effects; thus, via the cancellation of errors, the *D*_e_ results are good. Specifically, our B3LYP/aug-cc-PVQZ(-PP) values are in better agreement with the experimental ones, and the energy differences between experimental and calculated values range from 0.046 (OsB) to 0.410 eV (LaB), apart from TaB, where the calculated value overestimates the experimental *D*_0_ by 0.749 eV. On the contrary, for the second-row MBs, the energy differences between experimental and calculated values range from 0.002 eV to 0.343 eV. Finally, note that the main aim of the present study is to study the periodic bonding schemes and trends for MB molecules over three rows, providing a quantitative and qualitative picture of the chemical bonding in the MB species, which is presented here adequately.

## 4. Computational Details

The ground states of the ZnB and the second- and third-row-transition-metal monoborides, MBs, were calculated employing the B3LYP [55,65], MN15 [56], and TPSSh [57] functionals, along with the correlation-consistent basis sets of Dunning et al., i.e., the aug-cc-pVQZ-PP basis set for all M samples except La, and the aug-cc-pVQZ basis set for B [58,59,60,61]. For the La atom, the def2-QZVPPD basis set was used [62]. Additionally, low-lying excited states were calculated for the NbB, TcB, LaB, TaB, ReB, and HgB molecules. Bond lengths, dissociation energies with respect to the adiabatic products and with respect to ground state products, frequencies, and dipole moments were calculated. The charges were obtained via the natural population analysis, NPA. In the case of the NbB molecule, additional MRCISD (MRCISD+Q)/aug-cc-pVQZ(-PP) calculations were carried out to clarify the ground state. In their reference CASSCF calculations, the eight valence electrons were distributed in ten orbitals (5s4d + 2s2p), resulting in 2060 configuration state functions (CSFs), while the number of MRCISD CSFs were 5.9 × 10^7^ and they were reduced to 1.1 × 10^7^ after the internally contracted approach. All DFT calculations were performed using the GAUSSIAN package [66]. The multireference calculations were carried out using the MOLPRO package [67].

## 5. Summary and Conclusions

Boron plays an important role in chemistry, biology, and materials science. Diatomic transition-metal borides are important building blocks of many complexes and materials and thus the knowledge of their dissociation energy, bond distances, and bonding analysis dipole moments are very useful. It is interesting that boron forms a variety of orders of bonding from single to quadruple bonds and bonds of different types, i.e., covalent, dative, and ionic bonds. In the present paper, the diatomic borides of transition metals are reviewed and studied.

In the first part, a review on the available experimental and theoretical studies on the first-row-transition-metal borides, i.e., ScB, TiB, VB, CrB, MnB, FeB, CoB, NiB, CuB, and ZnB; the second-row-transition-metal borides, i.e., YB, ZrB, NbB, MoB, RuB, RhB, PdB, AgB, and CdB; and the third-row-transition-metal borides, i.e., LaB, HfB, TaB, WB, ReB, OsB, IrB, PtB, AuB, and HgB, is presented. There was a gap in the literature regarding TcB, which is studied here for the first time in detail. While where there were doubts regarding which state is the ground state for some MBs, here, it is clarified.

In the second part, the second- and third-row-transition-metal borides, MBs, are studied via DFT calculations using the B3LYP, TPSSh, and MN15 functionals in conjunction with the aug-cc-pVQZ-PP_M_/aug-cc-pVQZ_B_ basis sets. In the case of the NbB molecule, additional multireference calculations, i.e., MRCISD(MRCISD+Q)/aug-cc-pVQZ(-PP), were carried out to clarify which state is the ground state, because DFT could not decipher it. Bond distances, dissociation energies, frequencies, dipole moments, and natural NPA charges are presented. Comparisons between the MB molecules of all three rows and their bonding are presented. All differences and similarities are analyzed. Both result from the differences in bonding schemes. 

It was found that, apart from RhB which was recently reported to form quadruple bonds [2,4], RuB and TcB form quadruple bonds in their ground states as well. The X states of these three molecules present the same quadruple bonding, *σ*^2^*σ*^2^*π_x_*^2^*π_y_*^2^, and as the metal is escalated from Tc to Rh, the additional valence electron is added to the single occupied *d* orbitals, while the *X* states change from *X*^3^Σ^−^ (TcB) to *X*^2^Δ (RuB) and then to *X*^1^Σ^+^ (RhB). Finally, here, we studied the TcB molecule, i.e., three states were calculated, filling the gap that existed in the literature.

As a final remark, it has been reported that the states of the diatomic and triatomic molecules of sulfides are involved in complexes and solid or 2D materials as building blocks, explaining the variety of their morphologies [19,20]. Similarly, the present data may present a new approach to exploring the properties of solid state and 2D metastable polymorphic materials involving transition-metal borides, while they may assist in explaining the catalytic properties of complexes, including transition-metal boride bonds.

## Figures and Tables

**Table 1:** Previous theoretical and experimental data on the ground states of the 1st-row-transition-metal boride molecules, MBs (M = Sc, Ti, V, Cr, Mn, Fe, Co, Ni, Cu, and Zn): bond lengths *r*_e_ (Å), dissociation energies *D*_e_ (eV) and/or *D*_0_ (eV) with respect to the adiabatic products, vibrational frequencies *ω*_e_ (cm^−1^), anharmonic corrections *ω*_e_*x*_e_ (cm^−1^), and dipole moments *μ* (*μ*_FF_ = *δE*/*δε*) (Debye).

MB	Methodology	Ref.	State	*r*_e_	*D*_e_ ^a^	*D*_0_	*ω*_e_	*ω*_e_ *x*_e_	*μ* (*μ*_FF_)
**ScB**	icMRCISD/cc-pV5Z	[21]	*X*^5^Σ*^–^*	2.128	3.257	3.220	584.5	3.8	4.02(4.16)
	icMRCI+Q/cc-pV5Z	[21]		2.132	3.287	3.251	579	3.8	(4.23)
	icMRCISD+DKH+Q/cc-pV5Z	[21]		2.094	3.309	3.271	603	2.6	(4.13)
	B3LYP/6-311++G(3df)	[22]	*X*^5^Σ*^–^*	2.084	1.90 ^a^		612		3.95
	MRCI+Q	[23]				1.787 ^a^			
	Pauling method	[24]				2.8 ^a^			
	R2PI spectroscopy	[25]				1.72(6) ^a^			
**TiB**	icMRCISD/cc-pV5Z	[21]	*X*^6^Δ	2.077	2.723	2.687	587.6	3.5	3.13(3.42)
	icMRCI+Q/cc-pV5Z	[21]		2.080	2.797	2.761	583	3.5	(3.51)
	B3LYP/6-311++G(3df)	[22]	*X*^6^Δ	2.039	2.40	2.362	621		3.26
	Pauling method	[24]				<3.1			
	R2PI spectroscopy	[25]				1.956(16)			
**VB**	icMRCISD/cc-pV5Z	[21]	*X*^7^Σ^+^	2.039	2.268	2.231	589.9	3.75	2.73(3.20)
	icMRCI+Q/cc-pV5Z	[21]		2.043	2.381	2.344	585	3.8	(3.30)
	B3LYP/6-311++G(3df)	[22]	*X*^7^Σ^+^	2.011	2.17	2.132 ^a^	615		2.82
	R2PI spectroscopy	[25]				2.150(16) ^a^			
**CrB**	icMRCISD/cc-pV5Z	[21]	*X*^6^Σ^+^	2.166	1.141		420	18.5	2.05
	icMRCI+Q/cc-pV5Z	[21]	*X*^6^Σ^+^	2.183	1.353		405	20	1.43
	B3LYP/6-311++G(3df)	[22]	*X*^6^Σ^+^	2.187			415		2.41
**MnB**	icMRCISD/cc-pV5Z	[21]	*X*^5^Π	2.190	0.846	0.821	391.9	3.79	2.21(2.45)
	icMRCI+Q/cc-pV5Z	[21]		2.183	0.854	0.831	383	3.9	(2.39)
	B3LYP/6-311++G(3df)	[22]		1.828	1.27	1.232	621		2.47
**FeB**	R2PI spectroscopy	[26]	GS			2.43(2)			
	B3LYP/aug-6-311++G(3df)	[22]	*Χ*^4^Σ^−^	1.695	2.22		743		2.27
	icMRCISD/cc-pV5Z	[21]	*Χ*^4^Σ^−^	1.743	2.303		642.7	12.50	1.67(2.13)
	icMRCI+Q/cc-pV5Z	[21]	*Χ*^4^Σ^−^	1.747	2.346		645	13.20	(2.20)
**CoB**	R2PI spectroscopy	[26]	GS			2.954(3)			
	LIF spectroscopy	[27]	*Χ*^3^Δ_3_	1.705					
	B3LYP/aug-6-311++G(3df)	[22]	*Χ*^3^Δ	1.676	2.62		783		1.89
	icMRCISD/cc-pV5Z	[21]	*Χ*^3^Δ	1.696	2.736		756.9	6.14	1.03(1.83)
	icMRCI+Q/cc-pV5Z	[21]	*Χ*^3^Δ	1.700	2.849		757	6.10	(1.98)
**NiB**	R2PI spectroscopy	[26]	GS			3.431(4)			
	LIF spectroscopy	[28]	*Χ*^2^Σ^+^	1.698			778	4.90	
	B3LYP/aug-6-311++G(3df)	[22]	*Χ*^2^Σ^+^	1.676	2.83		793		1.66
	icMRCISD/cc-pV5Z	[21]	*Χ*^2^Σ^+^	1.676	3.239		805.1	3.93	0.80(2.16)
	icMRCI+Q/cc-pV5Z	[21]	*Χ*^2^Σ^+^	1.681	3.434		803	3.98	(2.41)
**CuB**	icMRCISD/cc-pV5Z	[21]	*Χ*^1^Σ^+^	1.934	1.839		496.9	5.54	1.21(1.71)
	icMRCI+Q/cc-pV5Z	[21]	*Χ*^1^Σ^+^	1.922	2.129		553	4.80	(1.62)
	Nonrelativistic CASPT2/PolMe	[29]	*Χ*^1^Σ^+^	1.910	2.806		518	4.25	
	No-pair DK CASPT2/NpPolMe	[29]	*Χ*^1^Σ^+^	1.865	2.354		563	4.34	
	No-pair DK CCSD(*T*)-20/NpPolMe	[29]	*Χ*^1^Σ^+^	1.909	1.522		555.0	4.33	
	R2PI spectroscopy	[30]	*Χ*^1^Σ^+^			2.26(15)			
	B3LYP/aug-6-311++G(3df)	[22]	*Χ*^1^Σ^+^	1.952	2.12		513		1.61
**ZnB**	B3LYP/aug-6-311++G(3df)	[22]	*Χ*^2^Π	2.274	0.370		286		1.70
	B3LYP/aug-cc-pVQZ(-PP)	^b^	*Χ*^2^Π	2.258	0.373	0.329	282.5		1.65
	TPSSh/aug-cc-pVQZ(-PP)	^b^	*Χ*^2^Π	2.217	0.573	0.529	322.7		1.84
	MN15/aug-cc-pVQZ(-PP)	^b^	*Χ*^2^Π	2.330	0.374	0.317	234.1		1.32

^a^ Dissociation energy with respect to the atomic ground state products. ^b^ This work.

**Table 2:** Previous theoretical and experimental data on the ground states of the 2nd-row-transition-metal boride molecules, MBs (M = Y, Zr, Nb, Mo, Tc, Ru, Rh, Pd, Ag, and Cd): bond lengths *r*_e_ (Å), dissociation energies *D*_e_ (eV) and/or *D*_0_ (eV), vibrational frequencies *ω*_e_ (cm^−1^), anharmonic corrections *ω*_e_*x*_e_ (cm^−1^), and dipole moments *μ* (*μ*_FF_ = *δE*/*δε*) (Debye).

MB	Methodology	Ref.	State	*r*_e_	*D*_e_ ^a^	*D*_0_	*ω*_e_	*ω*_e_ *x*_e_	*μ* (*μ*_FF_)
**YB**	R2PI spectroscopy	[25]				2.057(3)			
	DFT: B97-1/AVTZ-PP_Y_/VTZ_B_	[25]	*X*^5^Σ^−^	2.306	(1.99)	1.96	517		
	DFT: B3LYP/LANL2DZ	[34]	*S* = 2	2.254	2.17		582.4		4.65
	Pauling method	[24]				2.99			
**ZrB**	R2PI spectroscopy	[25]				2.573(5)			
	DFT: B97-1/AVTZ-PP_Zr_/VTZ_B_	[25]	*X*^6^Δ	2.159	(2.65)	2.61			
	DFT: B3LYP/LANL2DZ	[34]	*S* = 2.5	2.189	3.92		610.2		3.48
**NbB**	R2PI spectroscopy	[25]				2.989(12)			
	DFT: B97-1/AVTZ-PP_Nb_/VTZ_B_	[25]	^5^Π/^5^Φ	1.988	(3.11)	3.07	698		
	DFT: B3LYP/LANL2DZ	[34]	*S* = 1	1.996	3.40		662.7		3.84
	MRCISD+Q/aug-cc-pVQZ(-PP)	^b^	*X*^5^Π	2.018	2.901		708.9		3.10
	MRCISD+Q/aug-cc-pVQZ(-PP)	^b^	*A*^5^Φ	2.019	2.808		710.3		2.92
**MoB**	CASPT2/CASSCF/ANO-RCC-4ζ	[35]	*X*^6^Π	1.968	2.18		664		2.7
	DFT: B3LYP/LANL2DZ	[34]	*S* = 0.5	1.817	6.40		826		4.05
**TcB**	DFT: B97-1/(A)_Tc_VTZ_B_-(PP)_Tc_	[25]	*X*^5^Σ^−^			3.31			
**RuB**	R2PI spectroscopy	[26]				4.815(3)			
	Knudsen effusion	[36]	*X*^2^Σ	1.75		4.59(22)			
	LIF spectroscopy	[37]	^2^Δ_5/2_	1.7099					
	DFT: B3LYP/LANL2DZ	[34]	*S* = 0.5	1.761	6.48		910.8		3.49
**RhB**	R2PI spectroscopy	[26]				5.252(3)			
	Knudsen effusion	[36]	*X*^1^Σ	1.75		4.89(22)	915		
	LIF spectroscopy	[38]	*X*^1^Σ^+^	1.691(2)					
	MS-CASPT2/ANO-RCC-4ζ	[39]	*X*^1^Σ^+^	1.698					4.42
	LIF spectroscopy	[40]	*X*^1^Σ^+^	1.691(2)					
	MS-CASPT2/ANO-RCC-4ζ	[41]	*X*^1^Σ^+^	1.694	(5.7)	5.6	924		4.54
	MRCISD+Q/AV5Z-PP_Rh_ AV5Z_B_	[4]	*X*^1^Σ^+^	1.6873	5.473	5.414	938.3	4.32	(3.160)
	RCCSD(T)/AV5Z-PP_Rh_ AV5Z_B_	[4]	*X*^1^Σ^+^	1.6872	5.490	5.431	942.1	3.78	(2.865)
	ADF/PBE/TZ2P	[2]	*X*^1^Σ^+^		5.27				
	DFT: TPSSh/AVQZ-PP_Rh_ AVQZ_B_	[2]	*X*^1^Σ^+^	1.685					
	CCSD(T)/AVQZ-PP_Rh_ AVQZ_B_	[2]	*X*^1^Σ^+^	1.689					
	MRCI/AVQZ-PP_Rh_ AVQZ_B_	[2]	*X*^1^Σ^+^	1.687					
	MCSCF/Sapporo-(DKH)_Rh_-TZP	[42]	*X*^1^Σ^+^	1.701	5.165				
	DFT: B3LYP/LANL2DZ	[34]	*S* = 0	1.745	4.96		932.7		2.84
**PdB**	ESR spectroscopy	[43]	*X*^2^Σ						
	UHF/STO-3G*	[43]		1.608					
	LIF spectroscopy	[44]	*X*^2^Σ^+^	1.7278			650		
	Knudsen effusion	[36]	*X*^2^Σ	2.00		3.37(22)			
	DFT: B3LYP/LANL2DZ	[34]	*S* = 0.5	1.856	3.33		725.6		1.44
**AgB**	Nonrelativistic CASPT2/PolMe	[29]	*X*^1^Σ^+^	2.258	1.248	1.23	341	2.32	?
	No-pair DK CASPT2/NpPolMe	[29]	*X*^1^Σ^+^	2.098	1.684	1.66	425	3.41	?
	No-pair DK CCSD(*T*)-20/NpPolMe	[29]	*X*^1^Σ^+^	2.115	0.910	0.883	440	3.26	?
	DFT: B3LYP/LANL2DZ	[34]	*S* = 0	2.187	1.60		415.6		1.41
**CdB**	DFT: B3LYP/LANL2DZ	[34]	*S* = 0.5	2.668	0.22		198.3		1.67

^a^ Dissociation energy with respect to the atomic ground state products. ^b^ This work.

## Data Availability

All data are provided in the paper.
